# Loss of desmoglein-2 promotes gallbladder carcinoma progression and resistance to EGFR-targeted therapy through Src kinase activation

**DOI:** 10.1038/s41418-020-00628-4

**Published:** 2020-09-28

**Authors:** Sang-Hyun Lee, Jin-Man Kim, Dong Gwang Lee, Jangwook Lee, Jong-Gil Park, Tae-Su Han, Hyun-Soo Cho, Young-Lai Cho, Kwang-Hee Bae, Young-Jun Park, Seon-Jin Lee, Moo-Seung Lee, Yong-Min Huh, Deog Yeon Jo, Hwan-Jung Yun, Heung Jin Jeon, Nayoung Kim, Mina Joo, Jang-Seong Kim, Hyo Jin Lee, Jeong-Ki Min

**Affiliations:** 1 Biotherapeutics Translational Research Center, Korea Research Institute of Bioscience and Biotechnology (KRIBB), 125 Gwahak-ro, Yuseong-gu, Daejeon, 34141 Republic of Korea; 2 Department of Pathology, Cancer Research Institute and Infection Control Convergence Research Center, Chungnam National University College of Medicine, 266 Munhwa-ro, Jung-gu, Daejeon, 35015 Republic of Korea; 3 Research Center for Metabolic Regulation, Korea Research Institute of Bioscience and Biotechnology (KRIBB), 125 Gwahak-ro, Yuseong-gu, Daejeon, 34141 Republic of Korea; 4 Environmental Disease Research Center, Korea Research Institute of Bioscience and Biotechnology (KRIBB), 125 Gwahak-ro, Yuseong-gu, Daejeon, 34141 Republic of Korea; 5 Department of Biochemistry & Molecular Biology and Department of Radiology, Yonsei University College of Medicine, 50 Yonsei-ro, Seodaemun-gu, Seoul, 03722 Republic of Korea; 6 Department of Internal Medicine, Cancer Research Institute and Infection Control Convergence Research Center, Chungnam National University College of Medicine, 266 Munhwa-ro, Jung-gu, Daejeon, 35015 Republic of Korea; 7 Department of Biomedical Science and Infection Control Convergence Research Center, Chungnam National University College of Medicine, 266 Munhwa-ro, Jung-gu, Daejeon, 35015 Republic of Korea; 8 Department of Biomolecular Science, KRIBB School of Bioscience, Korea University of Science and Technology (UST), 217 Gajeong-ro, Yuseong-gu, Daejeon, 34141 Republic of Korea

**Keywords:** Tumour biomarkers, Tumour-suppressor proteins

## Abstract

Gallbladder carcinoma (GBC) exhibits poor prognosis due to local recurrence, metastasis, and resistance to targeted therapies. Using clinicopathological analyses of GBC patients along with molecular in vitro and tumor in vivo analysis of GBC cells, we showed that reduction of Dsg2 expression was highly associated with higher T stage, more perineural, and lymphatic invasion. Dsg2-depleted GBC cells exhibited significantly enhanced proliferation, migration, and invasiveness in vitro and tumor growth and metastasis in vivo through Src-mediated signaling activation. Interestingly, Dsg2 binding inhibited Src activation, whereas its loss activated cSrc-mediated EGFR plasma membrane clearance and cytoplasmic localization, which was associated with acquired EGFR-targeted therapy resistance and decreased overall survival. Inhibition of Src activity by dasatinib enhanced therapeutic response to anti-EGFR therapy. Dsg2 status can help stratify predicted patient response to anti-EGFR therapy and Src inhibition could be a promising strategy to improve the clinical efficacy of EGFR-targeted therapy.

## Introduction

Gallbladder carcinoma (GBC) is a rare neoplasm, but the most common malignancy of the biliary tract. Surgical resection is the only curative treatment. However, over 85% of GBC cases are unresectable with advanced-stage disease at the time of diagnosis [1–3]. Moreover, the recurrence rate is high even after complete surgical resection. The current standard first-line therapy for patients with advanced biliary tract cancer (BTC), including GBC, is a combination of gemcitabine plus platinum-based agents. Although combination therapy showed better responses and overall survival rates than gemcitabine alone [4–7], the overall outcome is disappointing. Molecularly targeted approaches have been investigated in attempt to overcome the low clinical outcome of chemotherapy. Based on the findings that epidermal growth factor receptor (EGFR) is pivotal for cell growth in many cancers and is overexpressed in 67–100% of patients with BTC, EGFR inhibitors have been evaluated for treating advanced BTC patients. However, at least three randomized clinical trials showed no positive results to favor the addition of cetuximab or erlotinib to chemotherapy [8, 9]. In colorectal cancer, oncogenic KRAS mutations predict the lack of efficacy of cetuximab when combined with chemotherapy [10]. However, such findings might not directly translate to BTC cases, as no correlation was found between the mutation status and clinical outcomes [9]. Therefore, identifying the regulators inducing the aggressive features of BTC and elucidating the underlying molecular events of BTC progression are necessary for developing new therapeutic strategies. In this context, it is critical to understand the molecular carcinogenesis and key molecular pathways associated with GBC to establish individualized targeted molecular therapies.

Adherens junctions, such as adherence junctions and desmosomes, are vital for maintaining epithelial homeostasis and integrity [11–13]. The dysfunction of adherence junctions is reportedly linked to tumor progression. Although the role of desmosomes in carcinogenesis is relatively unknown, several lines of evidence have shown that desmoglein-2 (Dsg2) can function either as a tumor suppressor or an oncogene in a context-dependent manner. Dsg2 expression was upregulated in skin squamous cell carcinoma and head and neck cancer [14, 15]. In contrast, Dsg2 expression was downregulated in pancreatic cancer, melanoma cells, and diffuse-type gastric cancer [16–18]. However, the expression level of Dsg2 and its potential role in GBC progression have not yet been reported.

The ubiquitously expressed cSrc, a non-receptor tyrosine kinase, is the first discovered proto-oncogene that functions as a critical regulator of tumorigenesis and metastatic progression [19]. The cSrc phosphorylates clathrin, which is involved in internalizing multiple types of membrane receptors (including EGFR), to promote their internalization and to enhance the endosomal pool of activated receptors that continue to signal until they are degraded [20]. cSrc also interacts with focal adhesion kinase (FAK), which plays a key role in cancer cell proliferation, motility, invasiveness, and eventually metastasis by modulating the formation and turnover of focal adhesions [21]. However, it is well established that overexpression of wild-type cSrc is by itself weakly oncogenic [22]. In addition, several reports have shown that mutations leading to the constitutive activation of cSrc in human cancers are rare. The poor transformation potential of cSrc, coupled with the lack of mutational activation in human cancers, has clouded our understanding of the role of cSrc in the development, maintenance, and progression of cancer [23–27]. Although mutational activation of cSrc is rare in cancer, hyper-activation of cSrc may confer resistance to chemotherapeutic agents such as 5-FU, doxorubicin, and cisplatin [28–30]. Therefore, to improve the efficiency of cSrc-targeted chemotherapy, diagnostic markers capable of screening patients are needed.

In this study, we investigated the pathophysiological roles of Dsg2 and found that it functions as a tumor-suppressor protein in GBC. Loss of Dsg2 modulated EGFR internalization, which led to the resistance to EGFR blockade therapy through the regulation of cSrc activities. In addition, we clearly showed that Dsg2 is a novel diagnostic marker for selecting potential responders to cSrc-targeted chemotherapy, and targeting cSrc may represent a rational therapeutic approach for treating GBC patients that are resistant to EGFR-targeted therapies.

## Materials and methods

### Mouse models

Eight-week-old BALB/c *nu/nu* nude mice were purchased from Orient. Mice were housed under specific pathogen-free conditions and used according to the guidelines of the Laboratory Animal Care Committee of the Korea Research Institute of Bioscience and Biotechnology. JCRB1033 cells (shCtrl or shDsg2) (5 × 10^6^) were inoculated subcutaneously into the left flank of each mouse. Tumor growth was monitored at 2-day intervals by measuring the length (*L*) and width (*w*) of each tumor with a caliper and calculating the tumor volume as 0.523 × *L* × *w*^2^. The experimental liver-metastasis animal model was established by intrasplenic transplantation of tumor cells. Mice were anesthetized and their spleens were exteriorized through a left lateral flank incision. Approximately 2 × 10^5^ cells (shCtrl, shDsg2) in 50 μl of Hank’s balanced salt solution were injected into the spleen parenchyma using a 28-gauge needle. The peritoneum and skin were closed in two layers with metal clips. Mice were euthanized by CO_2_ inhalation 3 weeks after the implantation, at which time the livers were collected and analyzed to determine the surface tumor nodules and the percentage of metastasis incidence.

### Human tissue samples

Human GBC samples were obtained for staining purposes from the Chungnam National University Hospital (Dr. Jin-Man Kim, Department of Pathology). All samples were anonymized. Tumor samples were collected from tissue blocks used for routine pathological examination. Clinicopathological parameters and the survival data were obtained at the time of surgery and during follow-up, respectively, from medical record reviews. All protocols using human materials were approved by the ethics committee of Chungnam National University Hospital. Written informed consent was obtained from all patients.

### Cell lines, reagents, and chemicals

Human GBC cell line JCRB1033 was purchased from the Japanese Collection of Research Bioresources Cell Bank (Osaka, Japan) and human GBC cell line SNU308 was purchased from the Korea Cell Line Bank (Seoul, Korea). The JCRB1033 cells were cultured in Dulbecco’s modified Eagle’s medium (DMEM) supplemented with 10% fetal bovine serum (FBS) and 1% penicillin/streptomycin in a humidified incubator at 37 °C. The SNU308 cells were maintained in RPMI-1640 medium supplemented with 10% FBS and 1% penicillin/streptomycin in a humidified incubator at 37 °C. The antibodies, chemicals, and reagents used in our study were listed in Supplementary Table S1.

### Immunohistochemistry and immunofluorescence

Four-micrometer-thick paraffin-embedded sections were prepared for immunohistochemical staining. For immunohistochemical staining of human and murine samples, the slides were first deparaffinized in xylene and then rehydrated in a graded alcohol series. Endogenous peroxidase activity was blocked by incubation with 0.3% hydrogen peroxide in phosphate-buffered saline (PBS) for visualization using the peroxidase reaction. Sections were washed in water before antigen retrieval was performed using a microwave to heat Tris-EDTA buffer (10 mM Tris-base, 1 mM EDTA, 0.05% Tween 20, pH 9.0) to 97 °C, in which the sections were incubated for 15 min. Slides were permeabilized in PBS containing 0.2% Triton X-100 (PBST) for 20 min and blocked for 30 min with 2% bovine serum albumin (BSA) and 2% normal goat serum (Vector Laboratories) in PBST. Primary antibodies and biotinylated secondary antibodies (Vector Laboratories) were diluted in blocking solution. Slides were incubated overnight at 4 °C with primary antibodies and for 1 h room temperature on the following day with secondary antibodies. Subsequently, the slides were incubated with avidin/biotin complex (Vector Laboratories) for 1 h and 3,3′-diaminobenzidine (Sigma) as a chromogen. Slides were counterstained with hematoxylin and mounted for viewing.

For immunofluorescence, murine sample slides were permeabilized in PBST for 20 min after antigen retrieval and blocked for 30 min with 2% BSA plus 2% normal goat serum (Vector Laboratories) in PBST. The slides were incubated overnight with appropriate primary antibodies. An Alexa Fluor 546-conjugated secondary antibody (Invitrogen) was used for staining. Slides were counterstained with DAPI and mounted with fluorescence mounting medium (DAKO).

For immunocytochemistry, cells were washed with PBS and fixed using 4% neutral buffered formalin. Cells were permeabilized in PBST for 20 min and blocked for 30 min with 2% BSA plus 2% normal goat serum (Vector Laboratories) in PBST. Primary antibodies were applied for 1 h and cells were incubated with secondary antibodies at room temperature for 1 h. Cells were counterstained with DAPI and mounted with fluorescence mounting medium (DAKO).

### TUNEL assay

TUNEL assays were performed with xenograft tumor tissue for immunohistochemical detection and quantification of apoptosis, based on labeling DNA strand breaks. The paraffin-embedded sections were first deparaffinized in xylene and rehydrated in a graded alcohol series. TUNEL staining was performed using the In Situ Cell Death Detection Kit, POD (Roche), according to the manufacturer’s recommended protocol.

### Evaluation of immunohistochemical staining

Immunohistochemical staining results were evaluated by two independent pathologists who were blinded to the patients’ clinicopathological details. The immunohistochemical staining results were categorized by classifying tumors into four grades, based on the staining intensity scores (0, no staining; 1, weak staining; 2, moderate staining; 3, strong staining). In the case of heterogeneous staining within samples, the higher score was selected if >50% of the cells showed the higher staining intensity. For all patients, scores from two tumor cores in the same patient were averaged to obtain a mean score.

### Short-hairpin RNA (shRNA) transfections

shDsg2 (NM_001943.1-987s1c1) and a non-targeting shRNA control (SHC002) encoded in the pLKO.1 lentiviral vector were purchased from Sigma. To generate stable transfectants, the lentiviral vector was co-transfected into Lenti-X-293T cells (Clontech, Shiga, Japan) with the virus packaging mix (Sigma) using the Lipofectamine 2000 reagent (Invitrogen, Carlsbad, CA, USA), according to the manufacturers’ instructions. The virus was incubated with GBC cells along with 5 μg/ml polybrene (Santa Cruz Biotechnology). After 20 h, the media were removed and replaced with fresh media containing 2 μg/ml puromycin (Santa Cruz Biotechnology). Puromycin-resistant clones were selected by culturing for 2 weeks in the presence of puromycin. Dsg2-expression levels were analyzed by western blot analyses.

### Flow cytometry analysis and cell sorting

Cells were incubated with a PE-conjugated anti-EGFR antibody or an unlabeled anti-cSrc antibody for 1 h in PBS containing 1% FBS. Cells were rinsed with the same buffer and further stained with a FITC-conjugated secondary anti-cSrc antibody for 1 h. Isotype-matched, conjugated antibodies were used for control staining. Cells were analyzed using an Amnis FlowSight flow cytometer (EMD Millipore, Billerica, MA, USA). Data analysis was performed using the FlowJo program.

For cell sorting, cells were dissociated using Accutase (STEMCELL Technologies) for 5 min and cells rinsed with using 3% BSA in PBS. Then, 1 µg of EGFR-PE antibody was added to a suspension of 1 × 10^6^ cells and incubated for 1 h at 4 °C. Next, the unbound antibody was washed away three times using the same buffer. Cells were sorted with a FACSAria II instrument (BD Bioscience) and used for subsequent experiments.

### In vitro cell-proliferation assay

Cell proliferation was determined using three different methods (MTT assays, single-cell colony-forming assays, and anoikis assays), as described below.

#### MTT assay

GBC cells were seeded (1 × 10^4^/well) in 96-well plates in 200 μl of DMEM or RPMI-1640 and cultured for the indicated time points (Fig. S3A). After incubation for 24–72 h, the MTT reagent (5 mg/ml) was added to the 96-well plates (5 μl/well) and incubation was continued for an additional 4 h. The MTT reaction was terminated by adding 100 μl DMSO to each well.

The effects of cetuximab and several kinase inhibitors (PP2, LY294002, PD98059, or dasatinib) on cell proliferation were examined in MTT assays. Briefly, cells were seeded in 96-well plates (1 × 10^4^ cells/well) in 200 μl of DMEM or RPMI-1640 and cultured overnight. Cells were pretreated with 40 μg/ml cetuximab, 10 μΜ dasatinib, or 40 μg/ml cetuximab, and 10 μΜ dasatinib in combination for 30 min, after which the cells were stimulated for 24–48 h with 50 ng/ml EGF (Fig. 6b) and/or several kinase inhibitors, and cultured for 48 h (Fig. 3d). After incubation for 24–48 h, the MTT reagent (5 mg/ml) was added to the 96-well plates (5 μl/well) and the incubations were continued for 4 h. The MTT reactions were terminated by adding 100 μl DMSO to each well. Cells cultured in DMEM or RPMI-1640 served as the control groups. The results were read by measuring the absorbance at 570 nm. Each experiment was performed in triplicate and repeated three times to assess for reproducibility of the results.

#### Single-cell colony-forming assay

GBC cells were seeded in 60 mm tissue culture plates at a density of 1 × 10^4^ cells/plate in 2 ml of DMEM or RPMI-1640 medium, and cultured for 72 h (Fig. 2a). The effects of cetuximab and dasatinib on cell proliferation were determined in colony-forming assays. Briefly, cells were seeded in 60 mm tissue culture plates (1 × 10^4^ cells/plate) in 2 ml of DMEM or RPMI-1640 and cultured overnight. Cells were pretreated with 40 μg/ml cetuximab, 10 μΜ dasatinib, cetuximab and dasatinib in combination for 30 min, and then the cells were stimulated for 72 h with 50 ng/ml EGF (Fig. 6c). After incubation for 72 h, the cells were fixed with 4% neutral buffered formalin and permeabilized with 0.2% Triton X-100 in PBS. The cells were then rinsed with PBS and stained using a 0.4% crystal violet solution for 2 h. After staining, the cells were rinsed with deionized water and incubated with DMSO. Empty tissue culture plates were stained with crystal violet to measure the background absorbance values. The cell-proliferation ratio was calculated as the experimental optical density (OD) value divided by the control OD value. The experiments were repeated three times.

#### Anoikis (anchorage-dependent cell death) assay

JCRB1033 cells (shCtrl and shDsg2) were seeded (1 × 10^6^ cells/well) in 2 ml of DMEM in six-well Ultra-Low Attachment culture plates (Corning) and cultured for 72 h. Subsequently, the cells were transferred back to adhesive tissue culture plates and photographed at 18 h later (Fig. 2e). The experiments were repeated three times.

### Transendothelial migration assay

Transendothelial migration of GBC cells through an 8-μm pore transwell chamber (Corning, Hickory, NC, USA) was assessed. The outer membrane was coated with 10 μl 0.2% gelatin, and the inside was coated with 10 μg/ml fibronectin. Human umbilical vein endothelial cells (HUVECs) were seeded in the upper wells at 2 × 10^4^ cells in 50 μl M199 medium (with 20% FBS) and cultured for ~48 h. After a HUVEC monolayer formed, GBC cells were stained with Cell-Tracker Green CMFDA (Invitrogen, Carlsbad, CA) for 30 min and then seeded on the HUVECs at a final density of 1 × 10^5^ cells in 50 μl medium without FBS. Growth medium containing 10% FBS was placed in the lower chamber, and the transwell chamber was incubated at 37 °C for 24 h. The cells remaining inside the transwells were removed by wiping with a cotton swab. The fluorescence intensities of the migrated cells were visualized using an Olympus IX81-ZDC inverted fluorescence microscope.

### Invasion assay

GBC cell invasion was examined in 8-μm pore transwell chambers (Corning Costar, Cambridge, MA). Briefly, the lower surface of the transwell was coated with 10 μg gelatin and the upper side was coated with 25 μg (0.5 μg/μl) reconstituted basement membrane substance (Matrigel; BD Biosciences). Next, fresh medium containing 10% FBS was placed in the lower chamber as a chemoattractant. GBC cells were incubated for 24 h in a medium containing 1% FBS, trypsinized, and suspended at a final concentration of 1.5 × 10^5^ cells/ml in FBS-free medium. The GBS cell suspension (100 μl) was loaded into each of the upper wells, and the chamber was incubated at 37 °C for 24 h. Cells were fixed and subjected to H&E staining. Chemotaxis activity was quantified by counting the cells that migrated to the lower side of the filter with an optical microscope. The numbers of cells in eight random fields were counted for each assay. The experiments were repeated three times.

### Collective cancer cell migration

The random motility of cells was determined using specialized wound-assay chambers (Ibidi, Munich, Germany). Cell suspensions (100 μl) at a density of 5 × 10^4^ cells were seeded onto each side of an IBIDI 35-mm μ-dish with culture inserts for live-cell motility analyses. After growth for 24 h, the culture inserts were removed, and the cells were incubated with fresh culture medium. Dishes were transferred to a live-cell incubating chamber (Live Cell Instrument, Seoul, South Korea) at 37 °C under 5% CO_2_ on the stage of an inverted fluorescence microscope (Olympus, IX81-ZDC) with a U*PLSAPO* ×20 objective lens. Random cell motility was monitored over a 12-h period by capturing images every 15 min; data analysis was performed using the MetaMorph program, version 7.1 (Universal Imaging, Media, PA).

### Western blotting

Cells were washed in PBS and solubilized in RIPA buffer (50 Mm Tris-HCl, 150 Mm NaCl, 1% NP-40, 0.1% sodium dodecyl sulfate [SDS], 0.5% sodium deoxycholate) supplemented with proteinase and phosphatase inhibitor cocktails (GenDEPOT). The cell lysates were centrifuged for 10 min at 4000 rpm, 4 °C to remove cellular debris. The protein contents of the cells were determined, and the cellular lysates were separated by SDS-polyacrylamide gel electrophoresis and electro-transferred to polyvinylidene difluoride membranes. After blocking in TBST with 5% nonfat milk, the membranes were incubated overnight with primary antibodies at 4 °C, followed by incubation with a horseradish peroxidase-conjugated secondary antibody (1: 1000 dilution) for 1 h. Immunoreactive bands were visualized using an Enhanced Chemiluminescence Kit (Amersham) and detected using a ChemiDoc Imaging System (Bio-Rad).

### IP experiments

Cells were lysed in RIPA buffer supplemented with proteinase and phosphatase inhibitor cocktails. The cell lysates were centrifuged for 10 min at 4000 rpm, 4 °C to remove cellular debris. To preclear the cell lysates, 50 μl of normal serum was added to 500 μg of each cell lysate studied and mixed with 10 μl of an agarose bead slurry. The cell lysates were incubated at 4 °C under rotary agitation for 1 h. After preclearing, the cell lysates were centrifuged at 4000 rpm at 4 °C for 10 min, and the supernatant was kept for the IP experiments. The supernatants were incubated for 4 h with 2.5 μg of anti-Dsg2, anti-cSrc, anti-EGFR, or anti-IgG1 antibodies at 4 °C under rotary agitation. Next, 80 µl of protein G- or protein A-conjugated agarose beads were added, and the resulting mixtures were incubated for 8 h at 4 °C under rotary agitation. Immunoprecipitated samples were washed three times with RIPA buffer, eluted with 2× loading buffer at 95 °C for 10 min, and analyzed by western blotting.

### Statistical analysis

The chi-square test and linear by linear association were used to assess correlation between EGFR and Dsg2 expression and clinicopathological features. Overall survival was defined from date of surgery to the date of death from all causes. A survival curve was plotted using the Kaplan–Meier method and analyzed using the log-rank test. A Cox proportional hazards model analysis was performed to analyze the effects of EGFR expression on survival. *P* values < 0.05 were considered statistically significant. All analyses were conducted using SPSS version 17.0 software program (SPSS, Chicago, IL). Data from in vitro and in vivo experiments were expressed as the mean ± standard error of the mean (SEM). Differences between groups were analyzed using Student’s *t* tests. *P* values < 0.05 were considered statistically significant. Data are representative of at least three independent experiments.

## Results

### Localization of EGFR and its clinical relevance in GBC

**Fig. 1:**
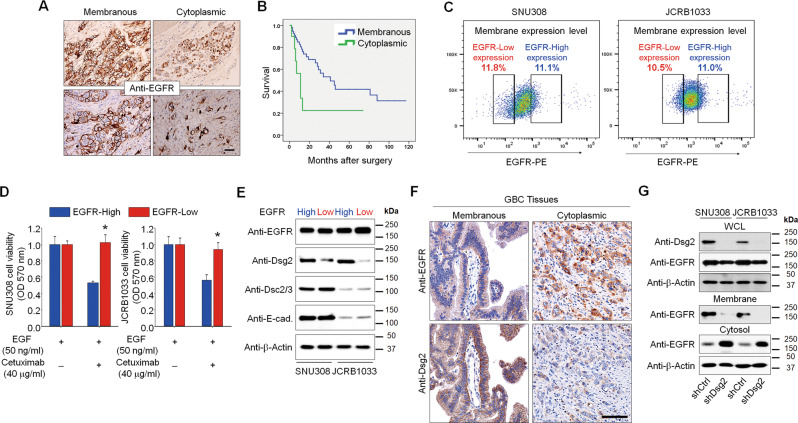
The expression level of Dsg2 might be involved in EGFR expression on the cell membrane. **a** Representative photomicrographs of immunohistochemical staining for EGFR in human tissues from patients with GBC. Scale bars: 100 μm. **b** The correlation between the EGFR expression pattern and survival rates in patients with GBC. Kaplan–Meier-based survival analysis for patients with membranous vs. cytoplasmic EGFR expression. **c** The isolation of GBC cells according to cell-surface EGFR expression using a FACSAria sorter, **d** cetuximab resistance in tumor cells with low EGFR expression. Cells expressing high or low levels of EGFR on the cell membrane were pretreated with 40 μg/ml cetuximab for 1 h, after which they were stimulated with 50 ng/ml of EGF for 48 h. Cell viabilities were assessed by MTT assays. *p < 0.05 compared with EGFR-high cells. **e** The expression levels of cell-junction cadherin proteins within EGFR-high and EGFR-low cell lysates were analyzed by western blotting using indicated antibodies. **f** Representative immunostaining of EGFR and Dsg2 in tissues of patients with GBC. Scale bars: 50 μm. **g** Western blot analysis of cytosolic and membrane protein fractions in shCtrl and shDsg2 cells. EGFR was dominantly expressed in the cytosolic fraction of both SNU308 shDsg2 and JCRB1033 shDsg2 cells.

Previous clinical trials with EGFR-targeted agents plus standard chemotherapy did not provide any clinical benefits for patients with GBC, even though EGFR is frequently overexpressed in GBC and is critical for gallbladder epithelial cell growth and proliferation [5, 8, 9]. To understand this, we first determined the expression and localization of EGFR in tissues from GBC patients by immunohistochemical staining. Among 67 tumor specimens from GBC patients, all were positive for EGFR even though EGFR is expressed mainly in the plasma membrane, 16% (11 cases) of EGFR-positive tumor specimens showed cytoplasmic EGFR expression (Fig. 1a). We examined correlations between EGFR localization and various clinicopathological parameters related to the malignancy of GBC (Supplementary Table S2). GBC patients with cytoplasmic EGFR expression tended to show more lymphatic invasion (90.9% vs. 60.7%; *P* = 0.054) and higher cellular grades (grade 3/4, 45.5% vs. 28.6%; *P* = 0.132) compared to those in patients with membranous EGFR (Supplementary Table S2). In addition, the patients with cytoplasmic EGFR showed significantly shorter survival times than those with membranous EGFR expression (median, 11 months vs. 41 months; *P* = 0.010) (Fig. 1b). Next, we performed univariate and multivariate analyses to investigate the clinical significance of various prognostic parameters affecting patient survival (Supplementary Table S3). Univariate analysis revealed that the pathologic T stage, nodal metastasis, differentiation, lymphatic invasion, and cytoplasmic expression of EGFR were significant risk factors affecting patient survival. Multivariate analysis indicated that the cytoplasmic expression of EGFR was an independent predictor of decreased overall survival in patients with GBC (hazard ratio, 3.617; 95% confidence interval, 1.354–9.658; *P* = 0.010).

Next, we tested whether the membranous and cytoplasmic expression patterns of EGFR might affect the treatment outcomes of anti-EGFR therapy and the acquisition of the malignant phenotype. GBC cell lines (SNU308 and JCRB1033) were separated based on their cell-surface EGFR expression levels (Fig. 1c and Fig. S1A). Cells were stimulated with EGF, and cell proliferation was measured in the presence or absence of cetuximab. Cetuximab treatment blocked the EGF-mediated proliferation of cells with high EGFR expression on the cell membrane (EGFR-high cells) but did not inhibit the proliferation of cells with low cell-surface EGFR expression (EGFR-low cells) (Fig. 1d). Furthermore, the EGF treatment in EGFR-high cells notably increased cell proliferation, but EGF did not show any significant effects in EGFR-low expression cells on the cell membrane (Fig. S1B). These results suggest that EGFR expression in the plasma membrane is a critical prerequisite for the efficacy of anti-EGFR therapies.

### The expression level of Dsg2 might be involved in EGFR expression on the cell membrane

Previous findings indicated that desmosomal cadherins regulate EGFR activity [31, 32] and that EGFR signaling induces ectodomain shedding of desmosomal cadherins and their subsequent internalization [33]. Thus, we investigated whether differences in EGFR localization were associated with the expression levels of various cadherin molecules in GBC cells. The expression levels of desmocollin 2/3 (DSC2/3) and E-cadherin were not significantly different between EGFR-low and EGFR-high cells. However, Dsg2 expression was markedly lower in EGFR-low cells than in EGFR-high cells (Fig. 1e). Moreover, Dsg2 expression was lower in the tissues of GBC patients with cytoplasmic EGFR expression (Fig. 1f and Fig. S2). To assess whether the loss of Dsg2 expression can influence EGFR localization, we generated two Dsg2-knockdown GBC cell lines (shDsg2) by lentivirus-mediated transduction of a Dsg2-specific shRNA (Fig. S3A). Immunofluorescence analysis clearly showed that Dsg2 is revealing at cell–cell borders in both shCtrl cells, whereas did not observe in shDsg2 cells (Fig. S3B). Total EGFR expression levels in whole-cell lysates were similar between shDsg2 cells and control (shCtrl) cells. However, EGFR expression was not detected in the membrane fraction, but was markedly increased in the cytoplasmic fraction of shDsg2 cells compared with shCtrl cells, suggesting that the loss of Dsg2 may mediate EGFR internalization (Fig. 1g).

### Loss of Dsg2 expression in GBC cells promotes tumor growth, cell motility, and invasiveness in vitro and in vivo

**Fig. 2:**
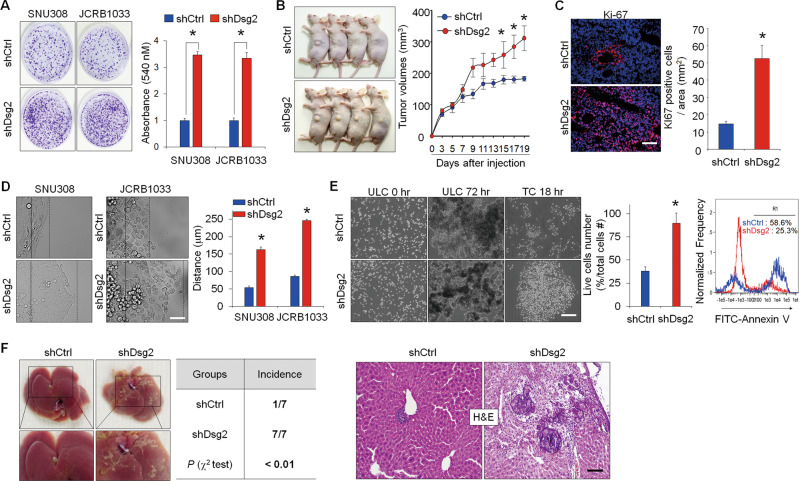
Dsg2 suppressed tumor progression in GBC. **a** GBC cells (1 × 10^4^) were seeded in 60-mm culture plates and cultured for 72 h. Subsequently, the cells were fixed and stained with crystal violet. The cells were solubilized in DMSO and the absorbance intensity was measured at 540 nM. Quantification of three independent assays is shown in the graphs. *p < 0.01 vs. each shCtrl cell. **b** shCtrl or shDsg2 JCRB1033 cells were subcutaneously injected into the left flank of BALB/c nude mice. *p < 0.05 compared with shCtrl. **c** Ki-67 staining of tumor xenograft sections. The representative bars indicate the number of Ki-67-positive cells per mm^2^. *p < 0.05 compared with shCtrl. Scale bars: 50 μm. **d** Cancer cell migration was analyzed by time-lapse microscopy. For each group, the motility of GBC cells was monitored over a 12-h period by capturing images every 5 min. The solid line indicates the starting position of cells and the dotted line indicates the end position of cells at 12 h. Quantification of the total distance between the first and last points relative to the total distance migrated is shown in the graph. *p < 0.05 compared with shCtrl. Scale bars: 50 μm. **e** Loss of Dsg2 expression in JCRB1033 cells notably suppressed anoikis-associated apoptosis. Representative bar and FACS results indicated the respective numbers of live cells and annexin V-positive cells during suspension in the ULC. ULC ultra-low attachment culture plates, TC tissue culture plates. *p < 0.05 compared with shCtrl. Scale bars: 200 μm. Error bars indicate ± SEM. **f** shCtrl or shDsg2 JCRB1033 cells were injected into the spleens of mice to test their liver-metastasis capacity. Representative images of the dissected liver with metastatic lesions (*n* = 7/group). H&E staining of liver sections from each group (right panel). Scale bars: 50 μm.

To investigate the biological role of Dsg2 in GBC cells, we examined the effects of Dsg2 silencing on the proliferation and motility of the SNU308 and JCRB1033 cell lines. Dsg2 knockdown significantly increased the proliferation of both cell lines in vitro (Fig. 2a and Fig. S4A). Next, to clarify the function of Dsg2 in GBC cells in vivo, we subcutaneously implanted shCtrl or shDsg2 JCRB1033 cells into nude mice. Mice implanted with shDsg2 cells showed significantly larger tumor volumes than mice implanted with control cells (Fig. 2b). Consistent with the in vitro cell-proliferation assay data, the numbers of proliferating cells in the tumor masses increased significantly in tumors derived from shDsg2 cells, compared to those derived from control cells, as assessed by immunohistochemical staining with a Ki-67 antibody (Fig. 2c). In addition, the migration of shDsg2 GBC cells was significantly higher than that of shCtrl cells (Fig. 2d and Supplementary Movies 1–4). To complete the multistep cascade of events involved in metastasis, cancer cells need to invade nearby parenchymal tissues and cross the endothelial cell lining. Cancer cells that acquired the anoikis-resistance phenotype can survive in the bloodstream and disseminate throughout the body via the circulatory and lymphatic systems. To address the effects of Dsg2 loss on the complex cascade of cancer metastasis, GBC cells were seeded on Matrigel, endothelial cell sheets, and ultra-low attachment culture plates to determine their abilities to undergo invasion, transendothelial migration, and anoikis, respectively. The shDsg2 cells showed significantly increased invasiveness and transendothelial migration compared to shCtrl cells (Fig. S4B, C). In addition, Dsg2 depletion appeared to make cells more resistant to anoikis, as the survival of shDsg2 cells was significantly higher than that of shCtrl cells (Fig. 2e). Consistent with in vitro results, liver metastasis substantially increased in mice implanted with shDsg2 cells compared to that in control mice at 3 weeks post injection (Fig. 2f). Collectively, these results strongly demonstrated that the loss of Dsg2 expression promoted growth and metastasis of GBC cells through increased cell motility and invasiveness.

To examine the clinical correlation between Dsg2 expression and the growth and progression of GBC, we examined Dsg2 expression in clinical tumor specimens from 67 patients by immunohistochemical staining. GBC cells showed diverse cytoplasmic and membrane staining patterns for Dsg2 (Fig. S5). Sixty cases (89.6%) showed positive staining according to an arbitrary scoring system (grade 1–3; grade 1, *n* = 23; grade 2, *n* = 15; grade 3, *n* = 22), and seven cases (10.4%) showed negative staining. Thirty-seven (55.2%; grade 2 and 3) and thirty (44.8%; grade 1 and negative) cases showed high and low Dsg2 expression, respectively. We examined correlations between Dsg2 expression and various clinicopathological parameters that are related to the prognosis of GBC patients (Supplementary Table S4). GBC patients with Dsg2-low expression showed a significant association with higher T stage (T3/4, 40.0% vs. 24.3%; *P* = 0.036) compared to those with Dsg2-high expression group. Furthermore, the Dsg2-low expression group showed more perineural (66.7% vs. 40.5%; *P* = 0.033) and lymphatic invasion (80.0% vs. 54.1%; *P* = 0.026). These data indicate that low expression of Dsg2 is strongly associated with the unfavorable clinical characteristics of GBC.

### Loss of Dsg2 expression activates Src kinase signaling in GBC cells

**Fig. 3:**
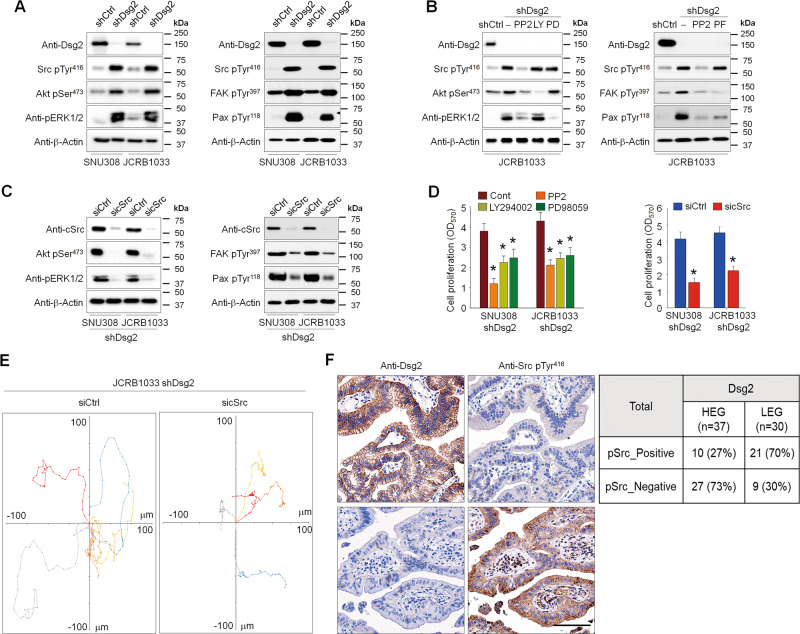
Loss of Dsg2 in GBC cells significantly increased Src kinase activation. **a** Phosphorylation of Src, Akt, ERK1/2, FAK, and Paxillin significantly increased in shDsg2 GBC cells. Cell lysates were analyzed by western blotting using the indicated antibodies. The pharmacological inhibition of Src kinase activity by PP2 (**b**) or RNA interference of cSrc mRNA by sicSrc (**c**) reduced Akt, ERK1/2, FAK, and Paxillin activation in shDsg2 GBC cells. Cells were treated with 10 μM PP2, 20 μM LY294002, 20 μM PD98059, or 20 μM PF-573228 for 30 min. GBC cells were transfected with 10 μM cSrc siRNA for 48 h, lysed, and subjected to western blot analysis with the indicated antibodies. **d** The inhibition of Src, PI3K, and MEK1/2 activity by the corresponding kinase inhibitors reduced cell proliferation (left panel). Knockdown of cSrc expression using cSrc siRNA significantly reduced GBC cell proliferation (right panel). Cell proliferation was assessed by MTT assays. *p < 0.05 compared with vehicle control (left panel) or siCtrl (right panel) cells. **e** Rose plots tracking the movement of five single siCtrl and sicSrc shDsg2 cells. Each color represents the track of an individual cell. **f** Representative images of IHC staining of Dsg2 and Src pTyr^416^ in clinical GBC samples. Scale bar: 50 μm.

To elucidate the molecular mechanism underlying Dsg2 loss-induced progression of GBC, we evaluated the effects of Dsg2 on the signaling pathways involved in cancer cell proliferation, survival, and migration. Dsg2 depletion substantially increased the phosphorylation of Src, Akt, ERK1/2, FAK, and paxillin in GBC cells (Fig. 3a). Next, to determine the regulatory mechanism of these signaling pathways, we treated Dsg2-knockdown cells with pharmacological inhibitors of PI3K (LY294002), MAPK (PD98059), Src family kinases (PP2), or FAK (PF-573228) and determined their effects on the respective signaling pathways. Treating Dsg2-knockdown cells with PP2 significantly decreased the phosphorylation of Akt at Ser^473^, ERK1/2, FAK at Tyr^397^, Paxillin at Tyr^118^, and Src at Tyr^416^, whereas treatment with LY294002, PD98059, and PF-573228 did not significantly affect signaling pathways other than the PI3K, MAPK, and FAK pathways, respectively (Fig. 3b). These data indicated that the loss of Dsg2 induced the Src-mediated activation of PI3K, MAPK, and FAK pathways. Furthermore, silencing of cSrc expression in Dsg2-knockdown cells substantially inhibited Akt, ERK1/2, FAK, and paxillin phosphorylation (Fig. 3c). Treatment with pharmacological inhibitors and silencing of cSrc expression by sicSrc significantly inhibited the proliferation and migration of both Dsg2-knockdown cells (Fig. 3d, e). Next, we assessed Src activation in tumor specimens from GBC patients with low or high expression of Dsg2. Among 30 GBC patient tissues with low expression of Dsg2, 70% (21 cases) of Dsg2-low tumor specimens showed cSrc activation. However, in 37 patient tissues with high expression of Dsg2, only 27% (10 cases) showed cSrc activation (Fig. 3f and Fig. S6). Collectively, these results clearly suggest that Dsg2 may suppress GBC progression by inhibition of Src tyrosine kinase activities.

### Dsg2 regulates Src kinase activity through binding of its Dsg2 IL domain to the cSrc SH2 domain

**Fig. 4:**
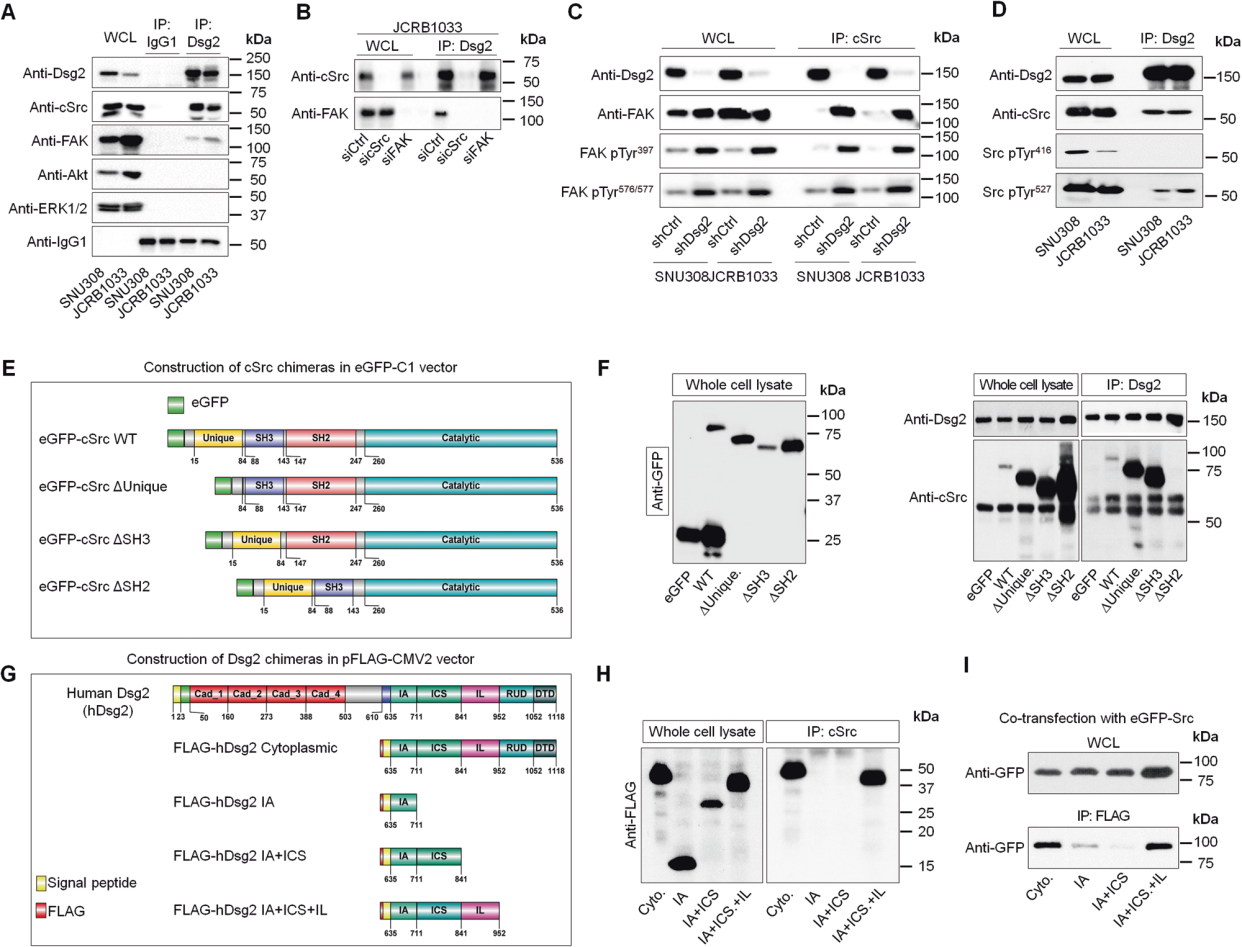
The Dsg2 IL domain directly bound the cSrc SH2 domain. **a** Immunoprecipitation (IP) and immunoblot (IB) analyses for Dsg2, cSrc, FAK, Akt, and ERK1/2 in GBC cells. WCL whole-cell lysates; IgG1 normal rabbit immunoglobulin G1. **b** The JCRB1033 cells were transfected with 10 μM cSrc or FAK siRNA for 48 h. Cell lysates were immunoprecipitated with an anti-Dsg2 antibody, blotted, and probed with an anti-cSrc or anti-FAK antibody. **c** GBC cell lysates IP with an anti-cSrc antibody and probing with indicated antibodies. **d** Co-IP analysis was performed to determine interactions between Dsg2 and Src pTyr^527^ in GBC cells. **e** Schematic diagram of the eGFP-tagged cSrc protein and the mutant constructs. **f** Western blot analysis of JCRB1033 lysates following IP with an anti-Dsg2 antibody after expressing the indicated constructs. Cell lysates were immunoprecipitated with an anti-Dsg2 antibody, blotted, and probed with an anti-cSrc antibody. **g** Diagram showing the strategy used to construct plasmids used to express FLAG-tagged hDsg2 truncation mutants. **h** Western blot analysis of the expressed hDsg2 variants using an anti-FLAG tag antibody (top left blot) and after lysate IP with an anti-cSrc antibody and probing with an anti-FLAG-tagged antibody (top right blot). **i** The eGFP-cSrc construct and each hDsg2 fragment were co-transfected into JCRB1033 cells. Western blots following anti-GFP IP of the indicated Dsg2 variants from cell lysates. Cyto cytoplasmic domain, IA intercellular anchor domain, ICS intercellular cadherin-like sequence domain, IL intercellular linker domain.

To further scrutinize the mechanism by which Dsg2 regulates cSrc activities, we investigate whether Dsg2 physically interacts with cSrc. Co-immunoprecipitation (Co-IP) experiments showed that Dsg2 interacted with cSrc and/or FAK, but not with Akt or ERK1/2 in GBC cells (Fig. 4a). To determine whether Dsg2 binds directly to cSrc and/or FAK, we knocked down cSrc or FAK expression using specific small-interfering RNAs (siRNAs) and performed immunoprecipitation experiments. In cells with cSrc silencing, FAK was not detected in Dsg2 immunoprecipitates, whereas FAK silencing did not influence the binding of Dsg2 to cSrc (Fig. 4b), indicating that Dsg2 directly binds to cSrc. Moreover, cSrc binding to FAK, especially to its active forms, increased significantly in Dsg2-depleted cells (Fig. 4c). Activation of cSrc proteins is tightly regulated by phosphorylation/dephosphorylation of different tyrosine residues—phosphorylation of Tyr^527^ inactivates cSrc, while phosphorylation of Tyr^416^ induces a conformational change in cSrc that activates the kinase domain. As shown in Fig. 4d, Dsg2 bound to the inactive (cSrc pTyr^527^) form more strongly than to the active form (cSrc pTyr^416^) in Dsg2-expressing GBC cells, implying that Dsg2 might bind to inactive cSrc (pTyr^527^) to maintain an inactive status, and that loss of Dsg2 might lead to activation of cSrc (cSrc pTyr^416^).

To determine Dsg2-binding domains in cSrc, we produced several GFP-tagged deletion mutants of cSrc (Fig. 4e) and determined the binding of each deletion mutant to Dsg2, after transiently expressing them in JCRB1033 cells. Intact cSrc and other deletion mutant forms were detected in Dsg2 immunoprecipitates, except SH2 domain-deleted cSrc mutant (Fig. 4f). Next, to determine which cytoplasmic domain of Dsg2 bound to cSrc, we also generated Dsg2 cytoplasmic domain constructs (Fig. 4g). Neither the intracellular anchor (IA) alone nor the IA with different intracellular cadherin-like sequences (ICSs) bound with cSrc, whereas, the recombinant intracellular linker (IL) with the IA and ICS clearly bound to cSrc (Fig. 4h). Furthermore, co-transfection with GFP-cSrc and each Dsg2 cytoplasmic domain construct showed that cSrc physically interacted with Dsg2 via the Dsg2-IL domain (Fig. 4i). These results indicated that Dsg2 directly interacted with cSrc through the Dsg2-IL domain and the SH2 domain of the inactive form of cSrc. These data provide insight into the mechanism by which Dsg2 regulates cSrc activity.

### Dsg2 loss-mediated cSrc activation induces EGFR internalization

**Fig. 5:**
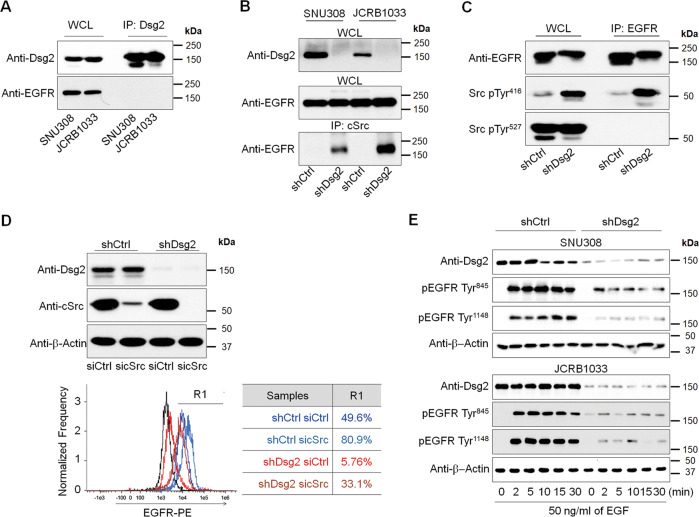
cSrc Activation Induced EGFR Internalization. **a** Co-IP was done with an anti-Dsg2 antibody. Whole-cell lysates (WCLs) and IPs of Dsg2 were analyzed by western blotting with an anti-EGFR antibody. **b** Western blot analysis of the shCtrl and shDsg2 GBC cell extracts after IP with an anti-cSrc antibody. Cell lysates were immunoprecipitated with an anti-cSrc antibody and probed with an anti-EGFR antibody. **c** Cell lysates were immunoprecipitated with an anti-EGFR antibody. Similar quantities of immunoprecipitates were analyzed by western blotting using EGFR, Src pTyr^416^, and Src pTyr^527^ antibodies. **d** shCtrl or shDsg2 JCRB1033 cells were transfected with 10 µM cSrc siRNA for 48 h, after which the cells were lysed and subjected to western blot analysis with the indicated antibodies (top blot). After 48 h, sicSrc- or siCtrl-transfected cells were fixed and labeled with a phycoerythrin (PE)-conjugated anti-EGFR antibody for FACS analysis (bottom panel). **e** shCtrl or shDsg2 GBC cells were stimulated with 50 ng/ml EGF for the indicated time points. Subsequently, the cells were lysed and western blotting was performed using antibodies against EGFR pTyr^845^ and EGFR pTyr^1148^.

To determine whether the reduction of EGFR expression on the cell membrane was a direct or indirect effect of the loss of Dsg2. We performed Co-IP experiments using whole-cell lysates with anti-Dsg2 antibodies and showed that EGFR did not bind to Dsg2 (Fig. 5a). However, the loss of Dsg2 substantially increased the interaction of EGFR with cSrc, preferentially with the active pTyr^416^ form of cSrc than with the inactive pTyr^527^ form in GBC cells (Fig. 5b, c). Next, we determined the effects of silencing Dsg2 or cSrc expression on EGFR localization by flow cytometry. Silencing Dsg2 expression significantly decreased the cell-surface expression of EGFR. However, silencing cSrc expression in both control and Dsg2-depleted cells increased the cell-surface expression of EGFR (Fig. 5d), and these effects were consistently observed in both EGFR-low and EGFR-high cells (Fig. S7). Moreover, silencing Dsg2 expression abrogated EGFR activation by epidermal growth factor (EGF; Fig. 5e). Collectively, these results suggest that Dsg2 may participate in EGFR signaling by modulating EGFR internalization. These data demonstrate that cSrc activation due to the loss of Dsg2 facilitated EGFR internalization in GBC cells and that cSrc inhibition may promote cell-surface EGFR expression.

### Inhibition of cSrc activity in Dsg2-downregulated GBCs suppresses tumor progression

**Fig. 6:**
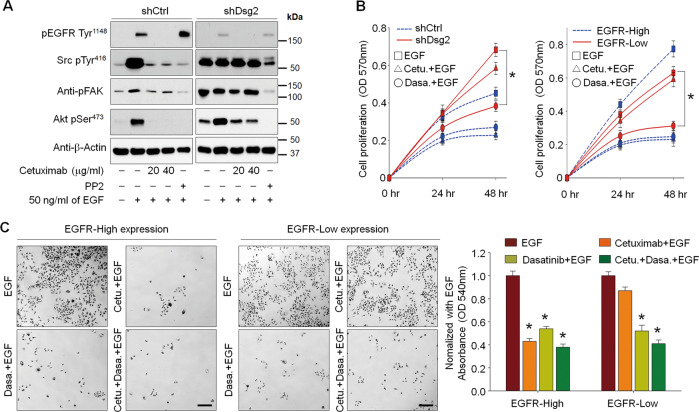
Inhibition of cSrc activity significantly reduced GBC cell proliferation. **a** Loss of Dsg2 expression in GBC cells increased resistance to cetuximab treatment. Cells were pretreated with 10 μM PP2, 20 μg/ml cetuximab, or 40 μg/ml cetuximab for 30 min and then stimulated with 50 ng/ml EGF for 15 min. Cell lysates were collected and analyzed by western blotting using the indicated antibodies. **b**–**c** Inhibition of Src kinase activity by the Src kinase inhibitor dasatinib notably reduced the proliferation of shDsg2 and EGFR-low cells. Cells were pretreated with 40 μg/ml cetuximab or 10 μM dasatinib alone (**b**, **c**) or co-pretreated with both cetuximab and dasatinib for 30 min (**c**). Subsequently, the cells were stimulated for the indicates times with 50 ng/ml EGF. Cell proliferation was assessed by performing MTT assays (**b**) and colony-forming assays (**c**); cells were stimulated with EGF for 72 h in the colony-forming assays (**c**). Quantification from three independent assays is shown in the graphs. *p < 0.01 vs. EGF; error bars indicate ± SEM. Scale bars, 100 μm.

**Fig. 7:**
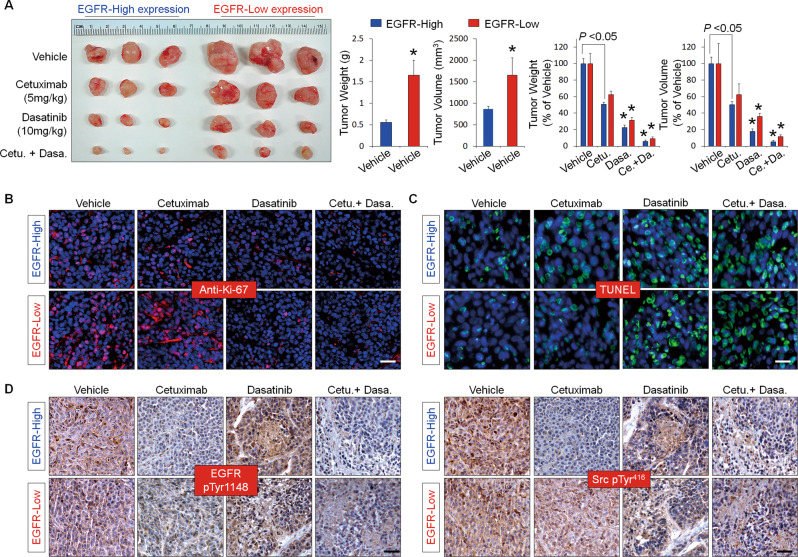
Dasatinib greatly reduced tumor growth. **a** Dasatinib treatment significantly reduced tumor growth. We isolated JCRB1033 cells according to cell-surface EGFR expression using a FACSAria sorter, and then each cell population was subcutaneously injected into the left flank of different BALB/c nude mice. For these experiments, the mice were treated as follows: cetuximab or IgG, 5 mg/kg intravenously twice per week; dasatinib, 10 mg/kg by oral gavage 2 days a week for 4 weeks. *p < 0.01 vs. vehicle. Representative images of Ki-67 (**b**) and TUNEL staining (**c**) in tumor xenografts sections. Scale bars in (**b**) 50 μm; in (**c**) 100 μm. **d** Immunohistochemical staining of EGFR pTyr^1148^ and Src pTyr^416^ in tumor xenografts sections. Scale bars: 50 μm.

Considering the role of cSrc in the surface localization of EGFR, we hypothesized that inhibition of cSrc activity could suppress EGFR internalization, which may improve the efficacy of EGFR-targeted therapeutics, including cetuximab. We found that EGF stimulation significantly increased the phosphorylation of Src, FAK, Akt, and ERK1/2 in control cells, but these proteins were not significantly activated in Dsg2-depleted cells. Pretreatment with cetuximab significantly inhibited EGF-induced phosphorylation of Src, FAK, Akt, and ERK1/2 in control cells, but not in Dsg2-depleted cells. In both types of cells, treatment with PP2 markedly decreased the phosphorylation of signaling molecules such as Src, FAK, Akt, and ERK1/2 (Fig. 6a). Next, we determined the effects of Src inhibition on the proliferation and viability of GBC cells in a clinically feasible setting using dasatinib, an approved cancer drug that targets Src family tyrosine kinases. In control and EGFR-high cells, treatment with cetuximab or dasatinib significantly reduced cell proliferation. However, while dasatinib also decreased the proliferation of Dsg2-depleted and EGFR-low cells in vitro, cetuximab did not (Fig. 6b). Consistently, although cetuximab did not affect the viability of JCRB1033 cells with low EGFR expression, dasatinib caused cytotoxicity, which was increased by co-treatment with cetuximab (Fig. 6c). To determine whether the effects of dasatinib or cetuximab on cell proliferation would translate in vivo, we subcutaneously implanted JCRB1033 cells with high or low cell-surface EGFR expression into nude mice. Mice implanted with EGFR-low cells had larger tumor volumes throughout the experiment than the mice with EGFR-high cells. Although cetuximab treatment significantly reduced tumor growth, dasatinib treatment showed a more significant reduction in tumor growth and the effects were more pronounced when combined with cetuximab treatment (Fig. 7a). Immunohistochemical staining of proliferating cells in tumor tissues showed notably lower numbers of EGFR-high cells in the cetuximab-treated group. This decrease was more significant in the dasatinib-treated group and most significant in the combined-treatment group with cetuximab plus dasatinib. In tumor tissues derived from EGFR-low cells, cetuximab treatment caused no significant changes in the numbers of proliferative cells. However, dasatinib treatment (alone or in combination with cetuximab) significantly inhibited cell proliferation (Fig. 7b and Fig. S8A). In contrast, the number of apoptotic cells significantly increased in both EGFR-high and -low tumor tissues after dasatinib treatment and more so after combination therapy with dasatinib plus cetuximab, as assessed by TUNEL staining (Fig. 7c and Fig. S8B). Cetuximab treatment slightly increased the number of apoptotic cells in tumor tissues with high EGFR expression, although a little difference was found in the number of apoptotic cells in the tumor tissues with low EGFR expression. In addition, EGFR activation, as determined by immunostaining with an anti-EGFR pTyr^1148^ antibody, was dramatically downregulated in tumor tissues treated with cetuximab alone or in combination with dasatinib. In contrast, EGFR activation was not changed by dasatinib treatment, compared with that in non-treated control tumor tissues. The effects of cetuximab on EGFR activation were more obvious in EGFR-high tumor tissues than in EGFR-low tumor tissues. Dasatinib treatment, alone or in combination with cetuximab, significantly abrogated the activation of Src in both EGFR-high and -low tumor tissues, compared with that in control tumor tissues (Fig. 7d). However, cetuximab treatment substantially abrogated Src activation in EGFR-high tumor tissues but did not affect Src activation in EGFR-low tumor tissues. Collectively, these results demonstrate that Dsg2 may be a useful theranostic marker for determining EGFR-targeted therapy as well as the Src activation status. Moreover, targeting Src may be a promising strategy for treating GBC patients with cytoplasmic EGFR expression.

## Discussion

Recent technological advances in genome sequencing have made rapid genome-wide screening of genetic and epigenetic alterations possible for individual cancer patients. Accordingly, expectations regarding personalized cancer therapy (based on the mutational profiles of each patient) have been rapidly increasing. Thus, identifying therapeutic targets for individual cancer patients, developing molecularly targeted agents, and determining biomarkers to stratify patients suitable for such targeted treatment are important for the development of effective personalized cancer therapy. In this study, we demonstrated for the first time that the desmosomal component Dsg2 played a tumor-suppressive role in GBC cells, as its loss-induced key biological activities of cells, including proliferation, motility, invasion, and transendothelial migration in vitro, as well as GBC tumor growth and metastasis in vivo.

EGFR is highly expressed in tumor cells in patients with GBC and plays critical roles in the growth and progression of many solid tumors. Although several clinical trials to assess the efficacy and tolerability of standard chemotherapy plus erlotinib or cetuximab treatment in patients with advanced BTC including GBC have been conducted, these trials did not show any promising results regarding the therapeutic potential of EGFR inhibition, even though it was well tolerated [8, 9]. It has been suggested that tumor cells acquire resistance to EGFR inhibitors or antibodies through the following three different mechanisms—(1) EGFR mutations T790M or S492R; (2) activation of a bypass signaling pathway, such as HER2 upregulation or KRAS activation; and (3) impairment of a pathway that is essential for EGFR tyrosine kinase inhibitor-mediated apoptosis [34]. The reason why targeted therapy against EGFR does not show clinical benefits in GBC patients is not clear. In this study, we investigated whether the localization of EGFR in GBC cells, either on the plasma membrane or in the cytoplasm, might affect the therapeutic efficacy of EGFR-targeted therapy. The overall survival rates and clinic-pathological results of GBC patients indicated that lower cell-surface EGFR expression was associated with poor clinical outcomes. In addition, we found that GBC cells with a cytoplasmic EGFR expression were highly resistant to anti-EGFR therapy with cetuximab, compared to cells with membranous EGFR expression.

Cell-adhesion proteins can act as key players in cancer progression and metastasis, as well as cell-to-cell adhesion functions. Until now, the role of Dsg2 in cancer progression and its functional mechanisms have been reported to be somewhat different in different cancer types. Kamekura et al. reported that Dsg2 acts as an oncogenic driver in colon cancer [32]. In colon cancer, loss of Dsg2 leads to a compensatory increase in Dsc2 expression and the increased Dsc2 suppresses cell proliferation by inhibition of EGFR downstream signaling such as Src [32]. However, we did not find any altered expression levels of desmosomal components including Dsc2 in Dsg2-depleted GBC cells (Fig. S9). Overmiller et al. also showed that knockout of Dsg2 decreases EGFR expression and abrogates the activation of EGFR and cSrc in squamous cell carcinoma [14]. In contrast, downregulation of Dsg2 expression has been associated with poor prognosis and increased tumor progression in diffuse-type gastric and prostate cancer [18, 35]. Similarly, we observed in this study that lower Dsg2 expression was associated with a poor prognosis of patients, as perineural and lymphatic invasion were highly increased in patients.

Dsg2 is involved in tumor progression through the regulation of EGFR and Src kinase activity. The reduction of Dsg2 on the squamous cell carcinoma membrane increased the release of extracellular vesicles (EVs) containing EGFR and Src and that these EVs containing EGFR and Src can modulate the tumor microenvironment, a step critical for tumor progression [36]. Although this report suggested that Dsg2 could modulate EGFR and Src activity, they did not demonstrate how Dsg2 directly regulates the activation of these proteins. Thus, a detailed explanation of the mechanism by which Dsg2 regulates EGFR and Src activation is needed. In this study, we clearly showed that Dsg2 is a negative regulator of Src kinase activation through direct interaction between Dsg2-IL domain and cSrc SH2 domain. These properties, such as the scaffolding protein role of Dsg2, inhibit tumor progression by sustaining the inactivated form of Src kinase. We demonstrated here that the loss of Dsg2 expression in GBC cells induces EGFR internalization through the regulation of cSrc activities. The loss of Dsg2 expression was strongly associated with EGFR clearance from the cell membrane, but this clearance could be significantly blocked by suppressing Src activation and expression. Although the regulatory molecular mechanism remains to be elucidated, Dsg2 appeared to directly interact with cSrc, and we showed for the first time that the Dsg2-IL domain directly and preferentially bound to the inactive form of Src through its SH2 domain. Consequently, the loss of Dsg2 in GBC cells significantly increased Src activation and facilitated the clearance of EGFR from the plasma membrane, leading to resistance to EGFR-targeted therapy.

Many reports have shown that cSrc activation may confer resistance to anticancer chemotherapy and/or EGFR-targeted therapy. For instance, GBC with cSrc overexpression and hyper-activation showed resistance to cisplatin treatment, whereas downregulation of cSrc mRNA by microRNA-31 significantly mitigated cisplatin resistance [37]. Qin et al. demonstrated that Src activation can induce resistance to the EGFR inhibitor, gefitinib, by activating the Akt- and/or Erk-signaling pathways in gallbladder adenocarcinoma cells [38]. In addition, cSrc activation also contributed to resistance to erlotinib, a small-molecule inhibitor of EGFR, by activating the hepatocyte growth factor receptor in head and neck cancer [39]. Collectively, these data indicate that Src is a key signaling node of multiple resistance mechanisms to anti-RTK therapies. Consequently, targeting Src family kinases can dramatically enhance the therapeutic efficacy of anti-RTK drugs including trastuzumab, cetuximab, erlotinib, and sunitinib, indicating that inhibiting cSrc activity is a rational treatment strategy for cancers (such as GBC) resistant to EGFR-targeted therapy.

As Src family kinases are pleiotropic kinases, it is not surprising that aberrant activation of Src signaling contributes to diverse aspects of tumor development [40]. Several Src kinase inhibitors such as bosutinib, dasatinib, and ponatinib have been developed and approved as anticancer drugs by the United States Food and Drug Administration. However, the therapeutic efficacies of Src inhibitors as single agents in treating various solid tumors are not encouraging, due to the intrinsic complexity of Src signaling and the redundant pathways involved in tumor development [41–45]. Therefore, the development of diagnostic markers is urgently needed for the preselection of potential responders to anti-Src therapy to enhance clinical benefits. In this study, we showed that inhibition of cSrc activity by Src inhibitors (PP2 and dasatinib) or siRNA dramatically reduced GBC cell growth and motility in vitro and tumor growth in vivo. Thus, we strongly suggest that Dsg2 is a promising diagnostic marker for the selection of patients suitable for treatment with anti-Src therapy.

**Fig. 8:**
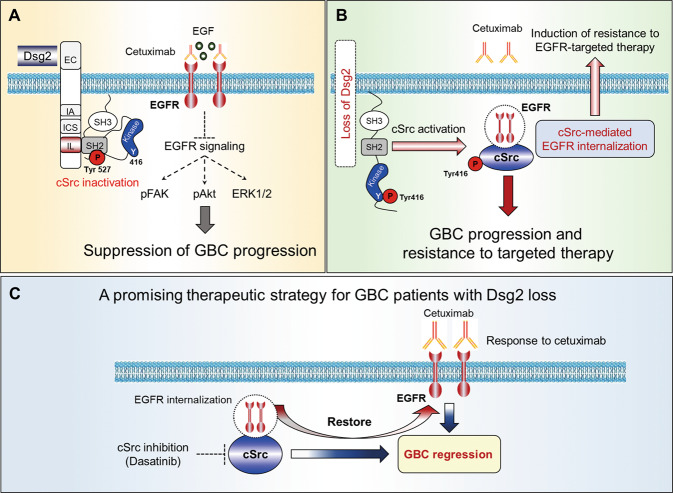
Proposed model by which Dsg2 suppresses tumor formation in GBC. **a** Dsg2 functions as a tumor suppressor through binding between the Dsg2 IL domain and cSrc SH2 domain. The EGFR-targeted therapy suppresses EGFR signaling pathway. **b** The loss of Dsg2 in GBC activates cSrc and promotes EGFR internalization, which induces resistance to EGFR-targeted therapy. **c** Combination therapy targeting both cSrc and EGFR is a promising therapeutic strategy for GBC patients with Dsg2 loss.

In summary, our data suggest a novel and missing link that connects the conventional cell-adhesive function of Dsg2 to tumor-suppressive function in GBC through the regulation of cSrc-mediated signaling pathways that play critical roles in EGFR clearance on the cell membrane, leading to acquired resistance to anti-EGFR therapies (Fig. 8). Our findings also indicate that targeting Src kinase activity is a promising therapeutic strategy for GBC patients with low level of Dsg2 and is potentially useful in overcoming resistance to current therapies, although further clinical studies are required to translate the current preclinical knowledge into clinical practice.

## Supplementary information

Supplementary Figure legends

Supplementary Figure 1

Supplementary Figure 2

Supplementary Figure 3

Supplementary Figure 4

Supplementary Figure 5

Supplementary Figure 6

Supplementary Figure 7

Supplementary Figure 8

Supplementary Figure 9

Supplemental Movie 1

Supplemental Movie 2

Supplemental Movie 3

Supplemental Movie 4

Supplementary Table S1

Supplementary Table S2

Supplementary Table S3

Supplementary Table S4
