# Supplementary material for: Loss of desmoglein-2 promotes gallbladder carcinoma progression and resistance to EGFR-targeted therapy through Src kinase activation
Source: Cell Death Differ. 2020 Sep 28;28(3):968–84. doi: 10.1038/s41418-020-00628-4 (PMC7937683; doi:10.1038/s41418-020-00628-4)
Supplement: Supplementary file 1 — Supplementary Figure legends [file 41418_2020_628_MOESM1_ESM.pdf]

Supplementary Figure Legends

Loss of Desmoglein-2 promotes gallbladder carcinoma progression and resistance to EGFR-targeted therapy through Src kinase activation

Running title: Dsg2 status is a Promising Theranostics Marker

Sang-Hyun Lee[1,†], Jin-Man Kim[2, †], Dong Gwang Lee[1†], Jangwook Lee[1], Jong-Gil Park[1], Tae-Su Han[1], Hyun-Soo Cho[1], Young-Lai Cho[3], Kwang-Hee Bae[3], Young-Jun Park[4], Seon-Jin Lee[4], Moo-Seung Lee[4], Yong-Min Huh[5], Deog Yeon Jo[6], Hwan-Jung Yun[6], Heung Jin Jeon[6], Nayoung Kim[7], Mina Joo[7], Jang-Seong Kim[1, *], Hyo Jin Lee[6, *], and Jeong-Ki Min[1,8, *]

Figure S1. A Heterogeneously expression of EGFR in GBC cells. GBC cells were isolated based on their cell-surface EGFR expression levels using a FACS Aria cell sorter. After 3 days, cells were stained with anti-EGFR antibody and these cells used for cetuximab-mediated cell cytotoxicity assay (Fig. 1d) and EGF effects on the cell proliferation (Fig. S1B). Scale bars, 20 μm. **B** EGF did not show any significant effects on the cell proliferation in EGFR-low expression cells. Cells were stimulated with EGF for indicated time points and measured cell proliferation by MTT assay. *p < 0.05 compared with vehicle.

Figure S2. Representative photomicrographs of Immunohistochemical staining for EGFR and Dsg2 in human gallbladder cancer tissues. **A** Membranous expression. **B** Cytoplasmic expression. Scale bars, 50 μm.

Figure S3. A Expression of Dsg2 level was determined by Western blotting. **B** Immunofluorescence staining with Dsg2 antibody. Scale bars, 20 μm.

Figure S4. Loss of Dsg2 significantly increased tumor progression. **A** Depletion of Dsg2 increased the growth of GBC cells, as determined by MTT assay. **B** Invasion assay were performed with the Transwell chamber. Scale bars, 200 μm. **C** Transendothelial migration assay. Cells were stained with cell-tracker and added to a HUVEC monolayer in the upper compartment. Scale bars, 200 μm. *p < 0.01 *vs.* each shCtrl cell.

Figure S5. Representative photomicrographs of immunohistochemical staining for Dsg2 in human gallbladder cancer tissues. **A,** No staining intensity. **B,** Weak staining intensity. **C,** Moderate staining intensity. **D,** Strong staining intensity. Scale bars, 100 μm.

Figure S6. Representative photomicrographs of Immunohistochemical staining for Dsg2 and Src $pTyr^{416}$ in human gallbladder cancer tissues. Dsg2 and Src pTyr416 expression were inversely correlated in tissue from GBC patients. **A** Dsg2, strong staining; Src pTyr416 negative staining. **B** Dsg2, weak or no staining; Src pTyr416 positive staining. Scale bars, 50 μm.

Figure S7. Suppression of cSrc expression significantly restored EGFR localization on the cell membrane. GBC cells were isolated based on their cell-surface EGFR expression levels. After 3 days, cells were transfected with 10 μM cSrc siRNA for 48 h. After 48 h, sicSrc- or siCtrl-transfected cells were fixed and labeled with a FITC-conjugated anti cSrc antibody or PE-conjugated anti-EGFR antibody for FACS analysis.

Figure S8. Dasatinib greatly reduced tumor cell proliferation and increased tumor cell cytotoxicity. **A** Quantification of number of Ki-67 positive cells in the tumor xenografts sections. The numbers of Ki-67 positive cells were counted from 9 images per each group. *p < 0.01 *vs.* EGFR-High Vehicle; $^{\#}$p < 0.01. Error bars indicate ± SEM. **B** Quantification of number of TUNEL positive cells in the tumor xenografts sections. The numbers of TUNEL positive cells were counted from 9 images per each group. *p < 0.01 *vs.* Cetuximab only. Error bars indicate ± SEM.

Figure S9. The expression level of desmosomal components. shCtrl and shDsg2 GBC cells immunoblotted for indicated antibodies.
