# Supplementary figures and images for: Loss of desmoglein-2 promotes gallbladder carcinoma progression and resistance to EGFR-targeted therapy through Src kinase activation
Source: Cell Death Differ. 2020 Sep 28;28(3):968–84. doi: 10.1038/s41418-020-00628-4 (PMC7937683; doi:10.1038/s41418-020-00628-4)

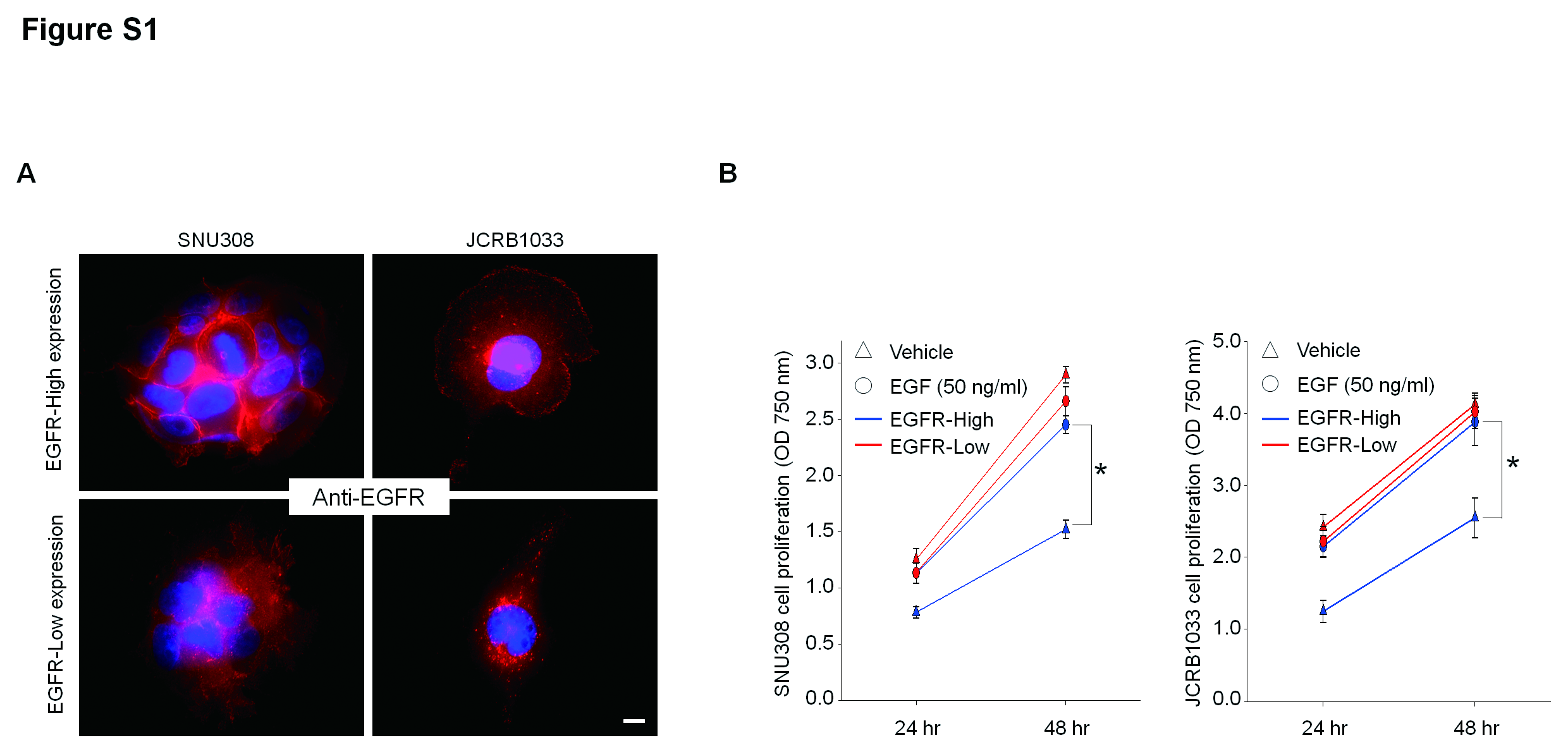

Supplement: Supplementary file 2 — Supplementary Figure 1 [file 41418_2020_628_MOESM2_ESM.tif]

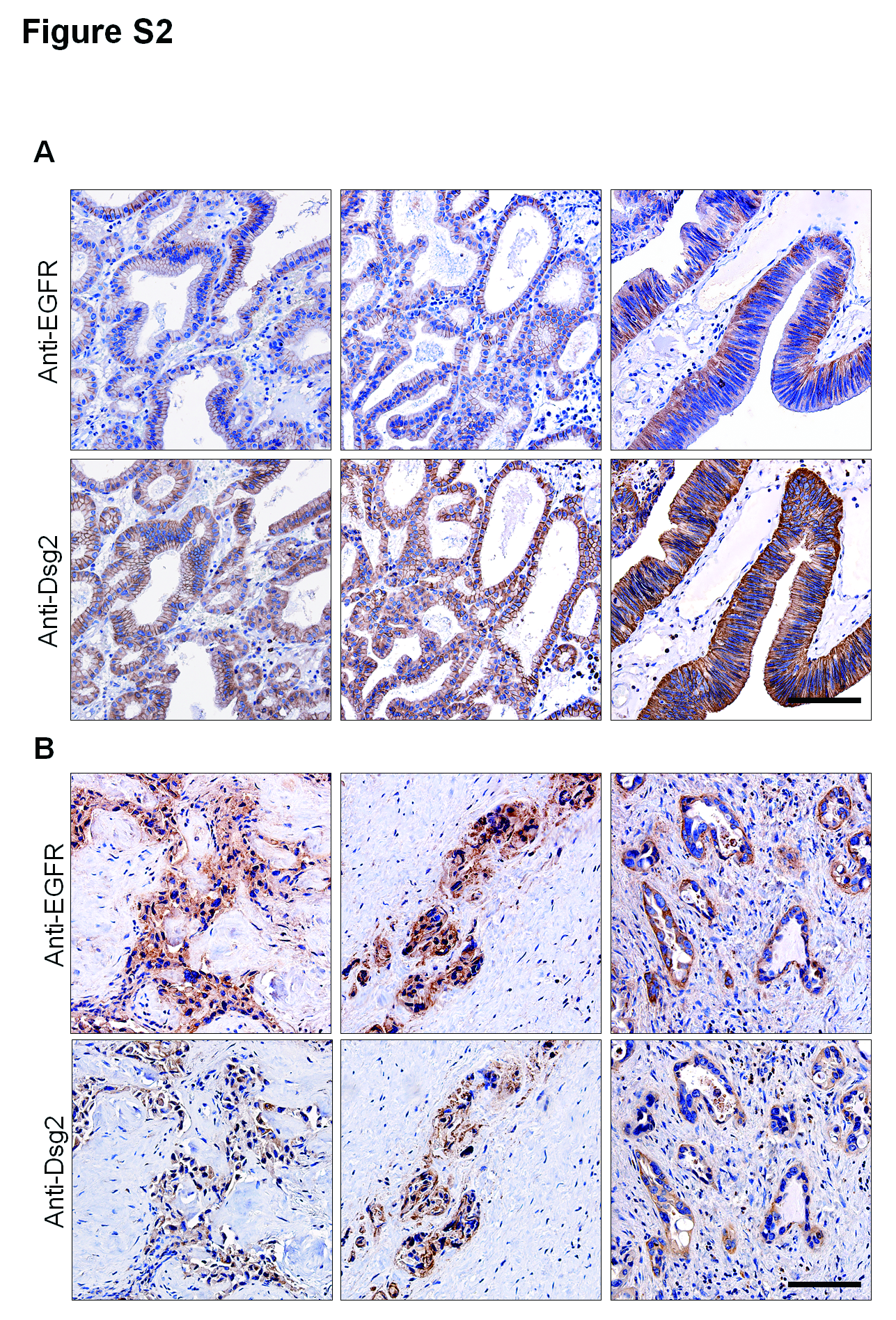

Supplement: Supplementary file 3 — Supplementary Figure 2 [file 41418_2020_628_MOESM3_ESM.tif]

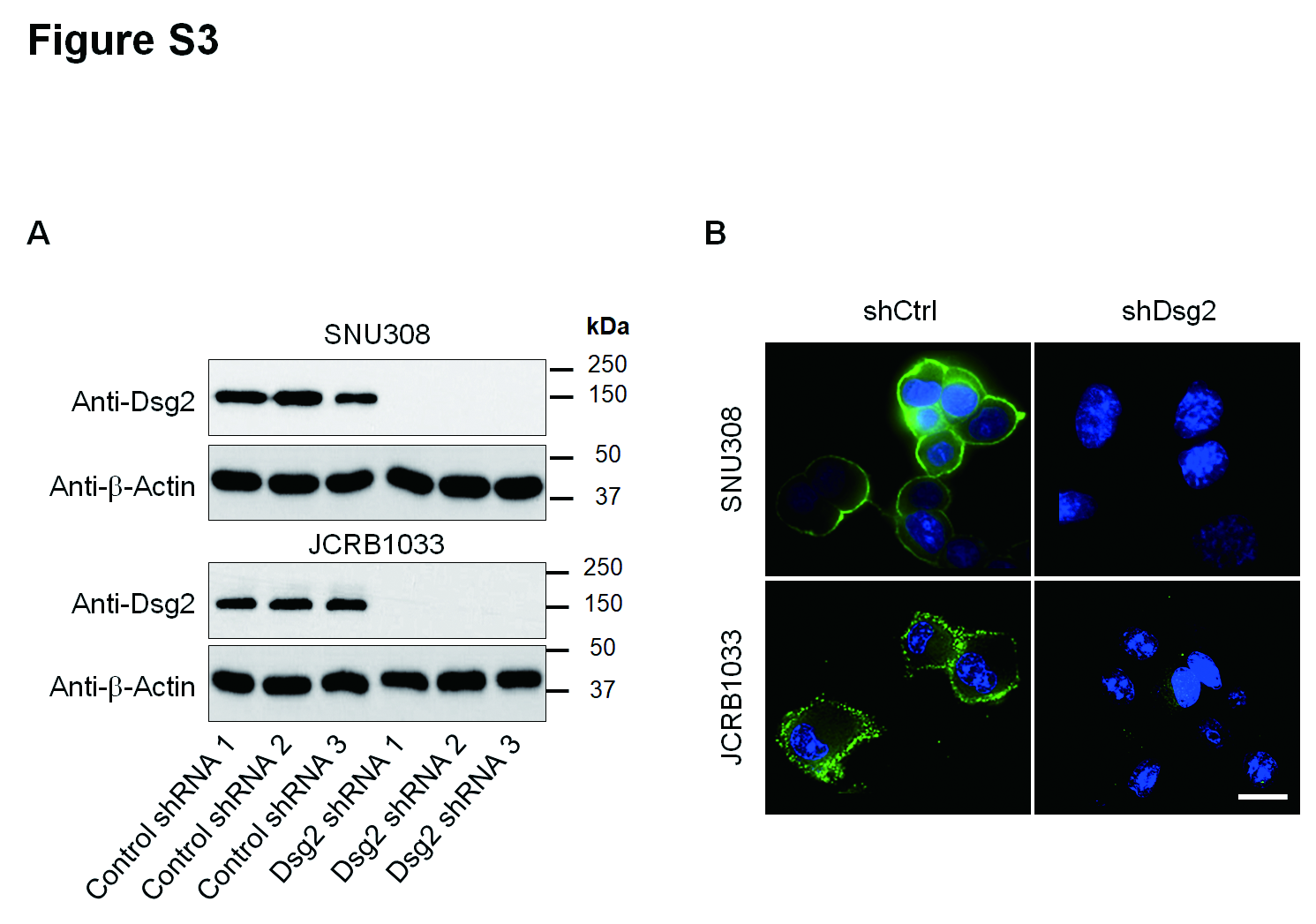

Supplement: Supplementary file 4 — Supplementary Figure 3 [file 41418_2020_628_MOESM4_ESM.tif]

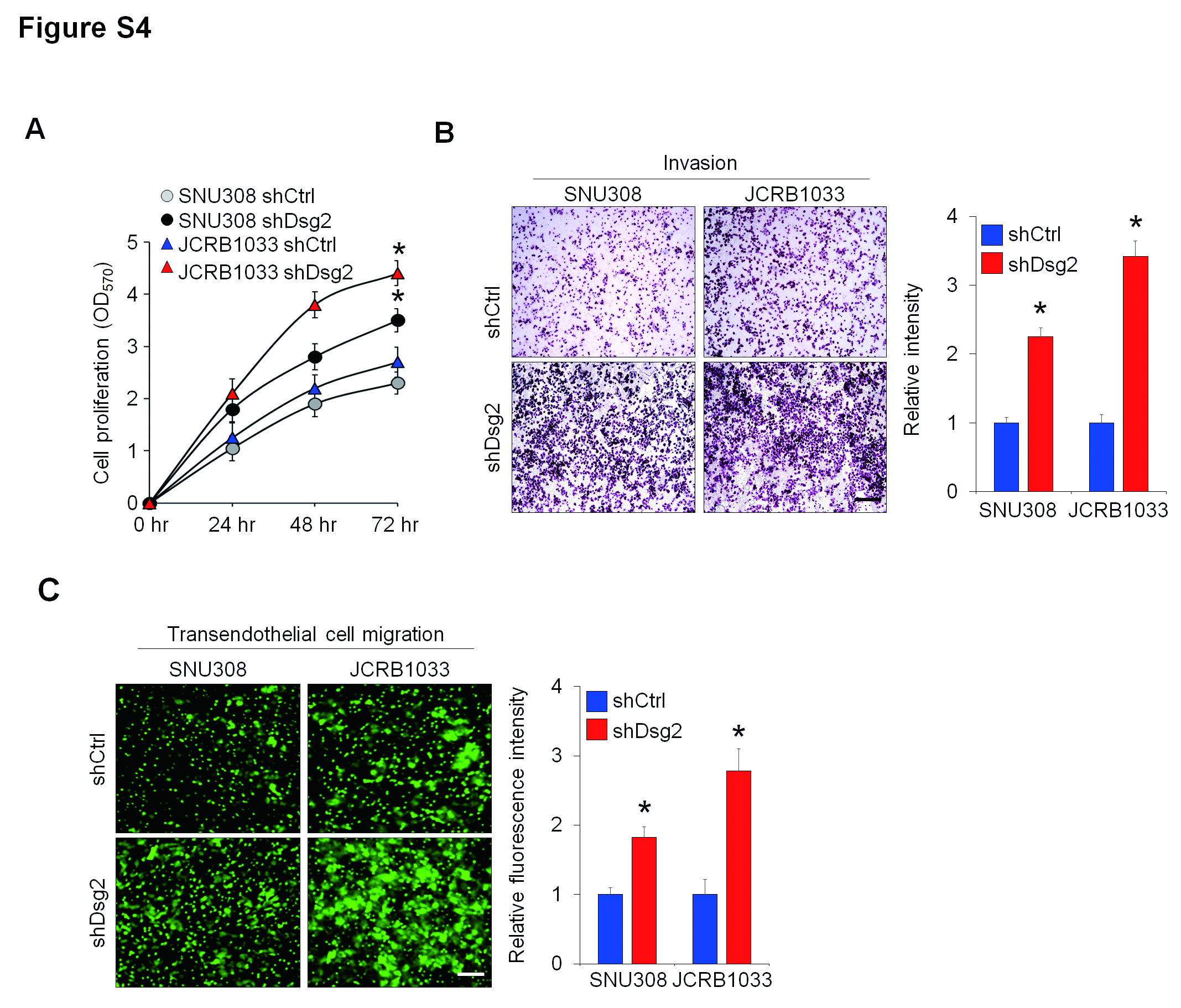

Supplement: Supplementary file 5 — Supplementary Figure 4 [file 41418_2020_628_MOESM5_ESM.tif]

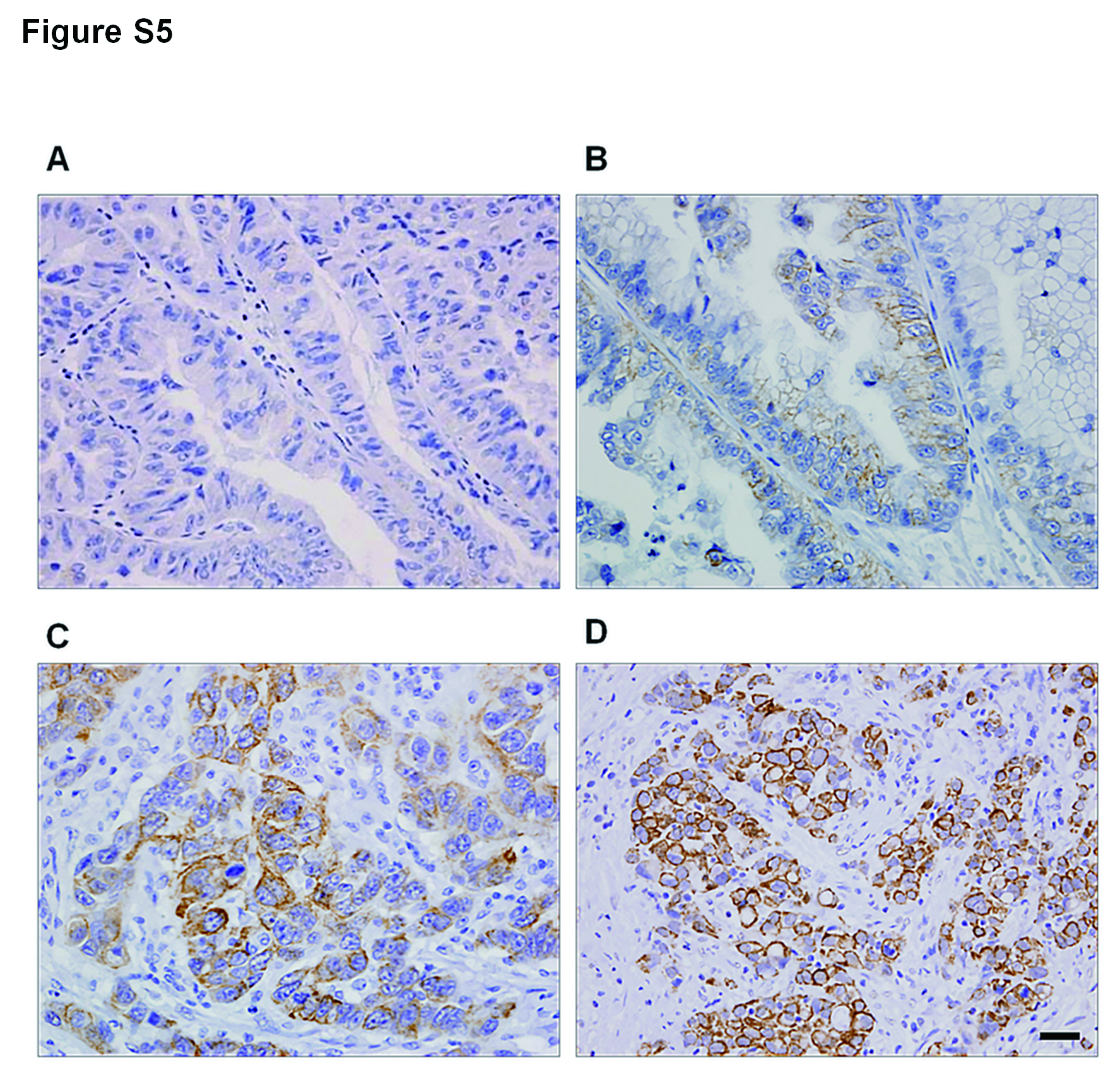

Supplement: Supplementary file 6 — Supplementary Figure 5 [file 41418_2020_628_MOESM6_ESM.tif]

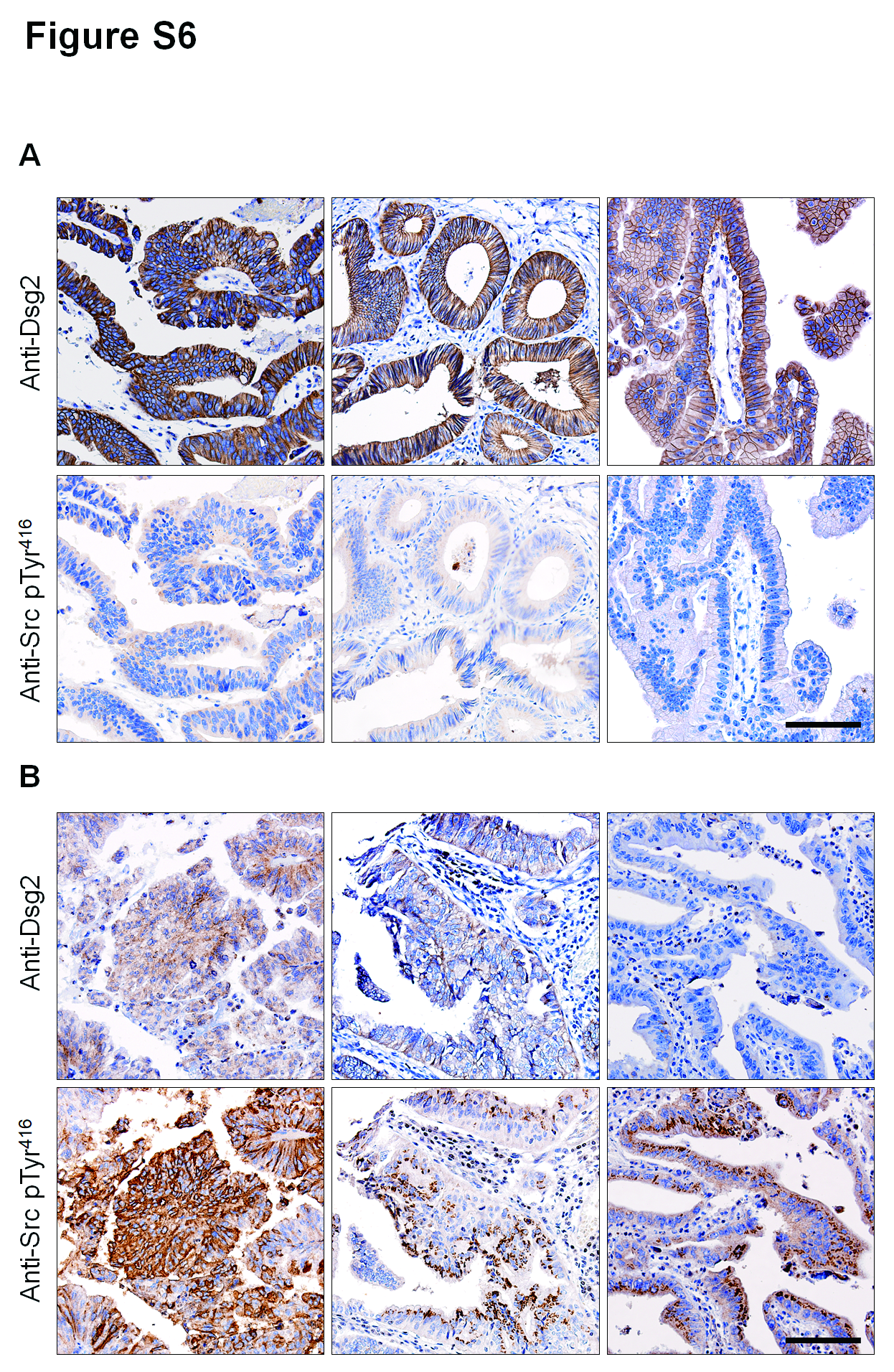

Supplement: Supplementary file 7 — Supplementary Figure 6 [file 41418_2020_628_MOESM7_ESM.tif]

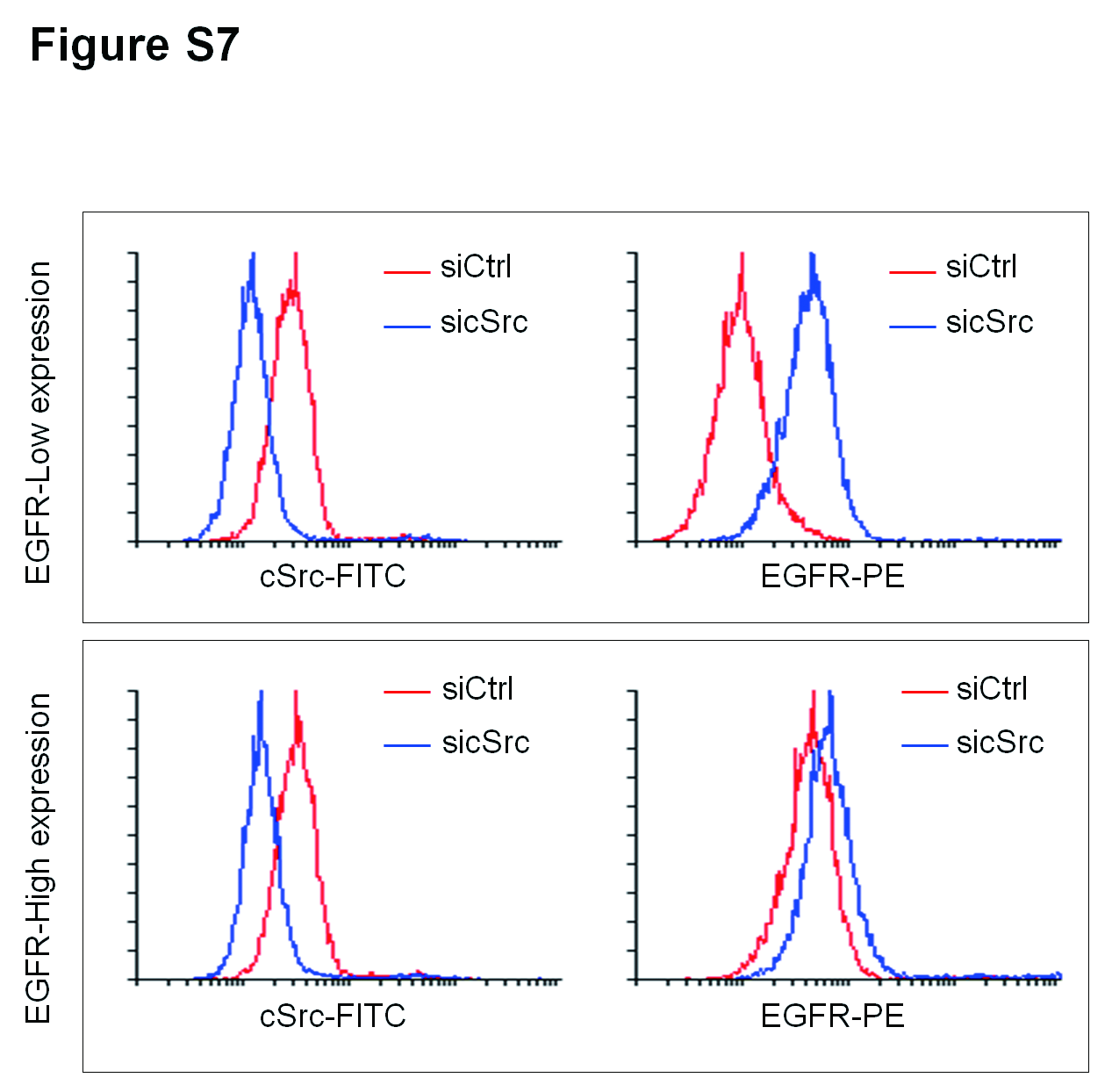

Supplement: Supplementary file 8 — Supplementary Figure 7 [file 41418_2020_628_MOESM8_ESM.tif]

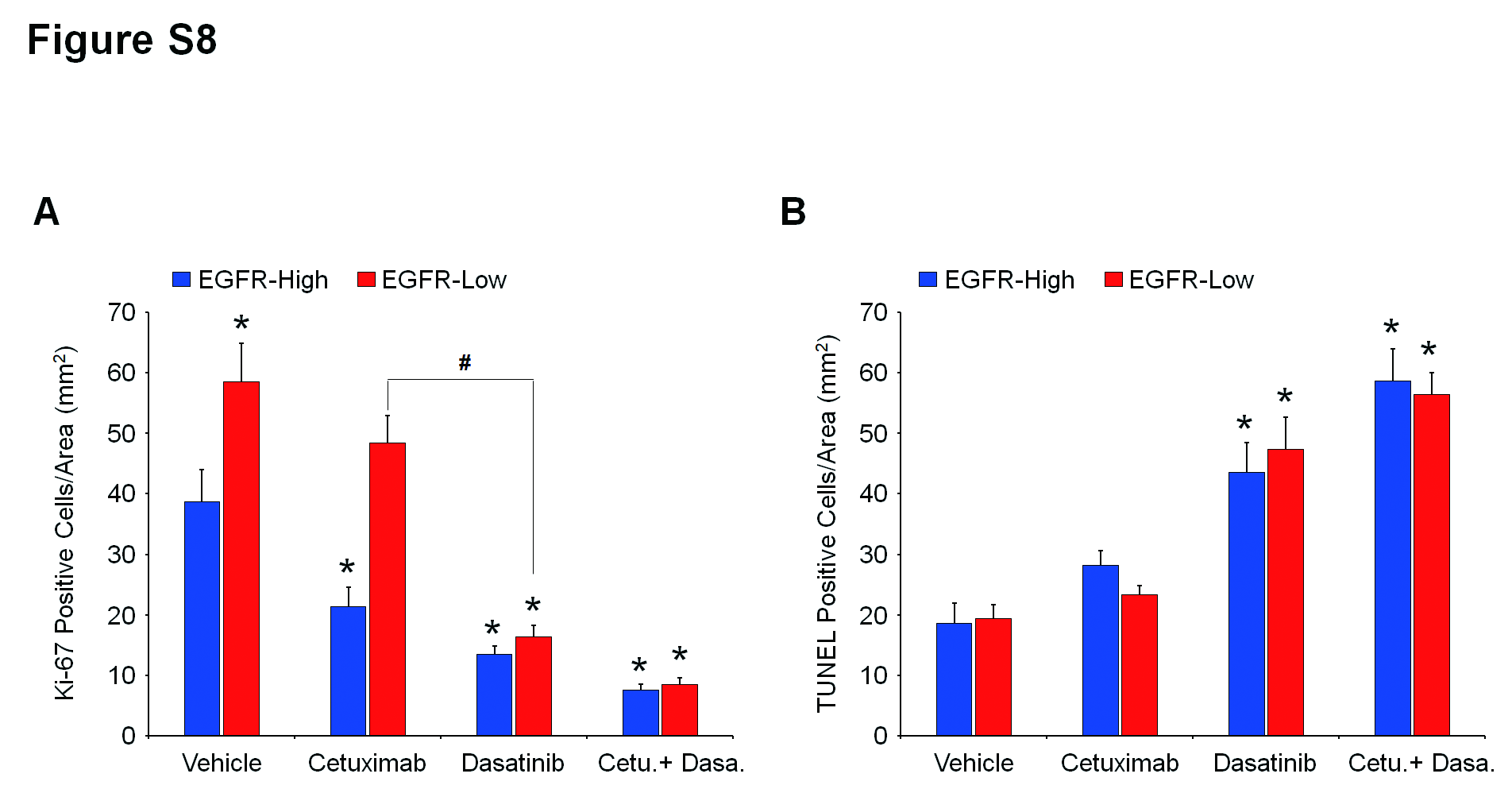

Supplement: Supplementary file 9 — Supplementary Figure 8 [file 41418_2020_628_MOESM9_ESM.tif]

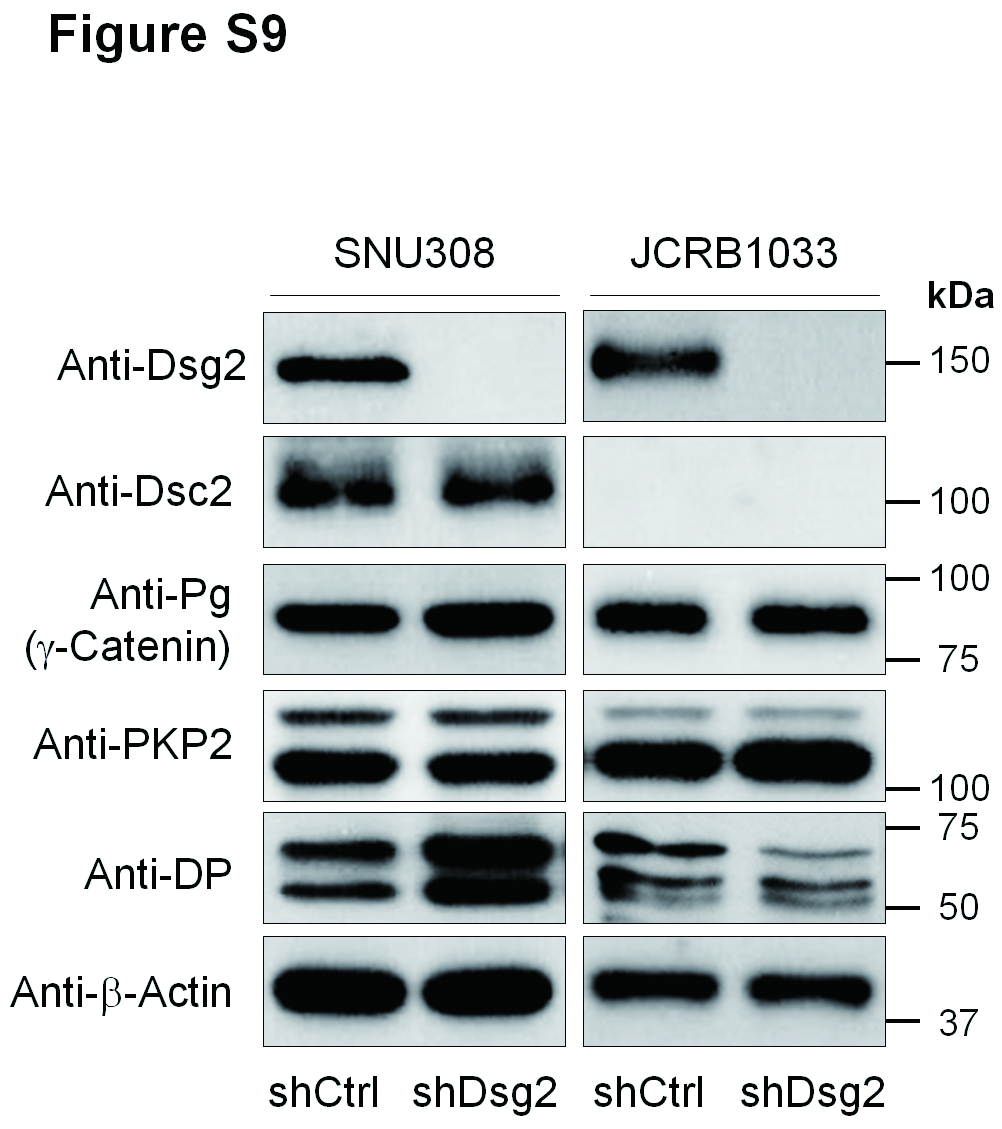

Supplement: Supplementary file 10 — Supplementary Figure 9 [file 41418_2020_628_MOESM10_ESM.tif]
