# Supplementary material for: Loss of desmoglein-2 promotes gallbladder carcinoma progression and resistance to EGFR-targeted therapy through Src kinase activation
Source: Cell Death Differ. 2020 Sep 28;28(3):968–84. doi: 10.1038/s41418-020-00628-4 (PMC7937683; doi:10.1038/s41418-020-00628-4)
Supplement: Supplementary file 15 — Supplementary Table S1 [file 41418_2020_628_MOESM15_ESM.pdf]

Supplementary Table S1

REAGENT or RESOURCE	SOURCE	IDENTIFIER
Antibodies		
EGF Receptor (D38B1) Antibody	Cell Signaling Technology	Cat#4267; RRID:AB_2246311
Phospho-EGF Receptor (Tyr845) Antibody	Cell Signaling Technology	Cat#2231S; RRID:AB_1264154
Phospho-EGF Receptor (Tyr1148) Antibody	Cell Signaling Technology	Cat#4404; RRID:AB_331127
EGF Receptor-PE (R-1) Antibody	Santa cruz	Cat#sc-101 PE; RRID:AB_627494
Desmoglein 2 (6D8) Antibody	abcam	Cat#ab14415; RRID: AB_2093427
DSC2/3 (3G130) Antibody	Santa cruz	Cat# sc-70994; RRID:AB_1122197
E-Cadherin (24E10) Antibody	Cell Signaling Technology	Cat#3195S; RRID:AB_10694492
FAK Antibody	Cell Signaling Technology	Cat#3285S; RRID: AB_2269034
Phospho-FAK (Tyr397) (D20B1) Antibody	Cell Signaling Technology	Cat#8556S; RRID:AB_10891442
Phospho-FAK (Tyr576/577) Antibody	Cell Signaling Technology	Cat#3281S; RRID:AB_331079
Paxillin (C-terminal) Antibody	ECM Biosciences	Cat#PP1161; RRID:AB_2253312
Phospho-Paxillin (Tyr118) Antibody	Cell Signaling Technology	Cat#2541; RRID:AB_2174466
Src Antibody	Cell Signaling Technology	Cat#2108S; RRID:AB_10695298
Phospho-Src (Tyr416) Antibody	Cell Signaling Technology	Cat#2101; RRID:AB_331697
Phospho-Src (Tyr527) Antibody	Cell Signaling Technology	Cat#2105S; RRID:AB_331034
Akt Antibody	Cell Signaling Technology	Cat#9272; RRID:AB_329827
Phospho-Akt (Ser473) Antibody	Cell Signaling Technology	Cat#9271; RRID:AB_329825
p44/42 MAPK (Erk1/2) (137F5) Antibody	Cell Signaling Technology	Cat#4695; RRID:AB_390779
Phospho-p44/42 MAPK (Erk1/2) (Thr202/Tyr204) (D13.14.4E) Antibody	Cell Signaling Technology	Cat#4370; RRID:AB_2315112
Beta Actin (C-term) Antibody	AbClon	Cat#abc-2004
Slug (C19G7) Antibody	Cell Signaling Technology	Cat#9585P; RRID:AB_10828257
FLAG M2 Antibody	Sigma-Aldrich	Cat#F1804; RRID:AB_262044
GFP Antibody	abcam	Cat#ab290; RRID:AB_303395
Anti-Human Ki-67 antigen	Dako	Cat#M7240; RRID:AB_2142367
Anti-Annexin V antibody (VAA-33) (FITC)	abcam	Cat#ab63556; RRID:AB_2057597
Erbitux (Cetuximab)	Merck	Cat#TAB-003; RRID:AB_2459632
Bacterial and Virus Strains		
psPAX2	addgene	Plasmid #12260
pMD2.G	addgene	Plasmid #12259

MISSION® pLKO.1-puro Empty Vector Control Plasmid DNA	Sigma-Aldrich	SHC001
pFLAG-CMV™-2 Expression Vector	Sigma-Aldrich	E7033
pEGFP-C1 Vector	BD Biosciences Clontech	Cat#6084-1
Biological Samples		
Paraffin-embedded gallbladder cancer tissue	Chungnam National University Hospital	Daejeon, Korea
Chemicals, Peptides, and Recombinant Proteins		
LY294002	Cell Signaling Technology	Cat#9901s; CAS:154447-36-6
PD98059	Cell Signaling Technology	Cat#9900s; CAS:167869-21-8
PP2	abcam	Cat#ab120308; CAS:172889-27-9
PF562271	abcam	Cat#ab141360; CAS:717907-75-0
Dasatinib	Selleckchem	Cat#S1021; CAS:302962-49-8
Recombinant Human EGF	R&D systems	Cat#236-EG; CAS:62253-63-8
Reagent or Resourse		
Trysine 0.05%	Gibco	Cat#25300054
DAPI solution	BD Pharmingen	Cat#564907
Crystal Violet	Sigma-Aldrich	Cat#c0775; CAS:548-62-9
Methylthiazolydiphenyl-tetrazolium bromide	Sigma-Aldrich	Cat#M5655; CAS:298-93-1
HBSS-Hank's Balanced Salt Solution	Gibco	Cat#14175079
Normal Goat Serum Blocking Solution	Vector Laboratories	Cat#S-1000
Matrigel Matrix	Corning	Cat#356234
Fetal Bovine Serum	Gibco	Cat#16140071
DMEM	Gibco	Cat#11995065
RPMI Medium 1640	Gibco	Cat#11875093
DPBS, no calcium, no magnesium	Gibco	Cat#14190250
Bovine Serum Albumin	GeneDEPOT	Cat#A0100-010
TritionX-100	Fisher Scientific	Cat#BP151-500
TWEEN 20	Sigma-Aldrich	Cat#P1379
Dimethyl Sulfoxide	Sigma-Aldrich	Cat#D8418
Penicillin Streptomycin	Gibco	Cat#15140122
Xpert Protease Inhibitor Cocktail Solution	GenDEPOT	Cat#P3100
Xpert Phophatase Inhibitor Cocktail Solution	GenDEPOT	Cat#P3200
Critical Commercial Assays		

Herculase Ⅱ Fusion Enzyme with dNTPs Combo	Agilent	Cat#600677
VECTASTAIN ABC HRP kit	Vector laboratories	Cat#PK-4000
3,3’-Diaminobenzidine tablets	Sigma-Aldrich	Cat#D4293
ECL Western Blotting Detection Reagents	Amersham	Cat#RPN2209
TUNEL assay kit	Roche	Cat#11684817910, Version 14
ANNEXIN V apoptosis kit	Abcam	Cat#ab14085
Experimental Models: Cell Lines		
Human Umbilical Vein Endothelial cells	Promo Cell	Cat#C-12200
SNU308	Korean Cell Line Bank	KCLB No. 00308
JCRB1033	Japanese Collection of Research Bioresources	JCRB No. JCRB1033
Experimental Models: Organisms/Strains		
Mouse: BALBc/nu/nu	ORIENTS,KOREA,SEOUL	BALB/c nude
Oligonucleotides		
H-DSG2-F-cloning-FlagV-not1 ATAAGAATGCGGCCGCGATGGCGCGGAGCCCGGGA	This paper	N/A
H-DSG2-R-cloning-FlagV-bamh1 GCGGATCCTTAGGAGTAAGAATGCTGTA	This paper	N/A
DSG2-IA-R-cloning-FlagV-bamh1 GCGGATCCCTCAGTGTAAGAGGCCGCTTT	This paper	N/A
DSG2-ICS-R-cloning-FlagV-bamh1 GCGGATCCTTGACCCAGGCAAACTTCAGC	This paper	N/A
DSG2-IPL-R-cloning-FlagV-bamh1 GCGGATCCGGTATTCTCTGAATTAACCAT	This paper	N/A
DSG2-sig-pep-reverse TCCACTTCCAACGTTAAAGC	This paper	N/A
DSG2-sig-TM-s GCTTTAACGTTGGAAGTGGACTGGGACCCG	This paper	N/A
DSG2-sig-TM-as GCAATTGCTGCGGGTCCCAGTCCACTTCCA	This paper	N/A
cSrc-cloning-pEGFP-C1-Full-F CCGCTCGAGCTATGGGTAGCAACAAGAGCAA	This paper	N/A
cSrc-cloning-pEGFP-C1-Full-R CCGGAATTCCTAGAGGTTCTCCCCGGGCT	This paper	N/A
cSrc-Deleltion-unique-F AGGATGCCAGCCAGCGGCGCGAGACAGACC	This paper	N/A

cSrc-Deleltion-unique-R TTGAAGGACAGGTCTGTCTCGCGCCGCTGG	This paper	N/A
cSrc-Deleltion-SH3-F CCGCTGGCCGGAGTGACCACCTACTACTCC	This paper	N/A
cSrc-Deleltion-SH3-R CGGCGTGTTTGGAGTAGTAGGTGGTCACTC	This paper	N/A
cSrc-Deleltion-SH2-F GCGCCCTCCGACTCCATCCATGCCCCACGT	This paper	N/A
cSrc-Deleltion-SH2-R TGCGGCTTGGACGTGGGGCATGGATGGAGT	This paper	N/A
Recombinant DNA		
DSG2 MISSION shRNA Bacterial Glycerol Stock	Sigma-Aldrich	SHCLNG-NM_001943; TRCN0000053843; NM_001943.1-
cSrc siRNA (h2)	Santa Cruz	Cat#sc-44250
Control siRNA	Santa Cruz	Cat#sc-37007
Software and Algorithms		
FlowJo 8.8.6	FlowJo	RRID: SCR_008520
Metamorph 7.1	Molecular Devices	https://www.moleculardevices.com/systems
