# Supplementary material for: Loss of desmoglein-2 promotes gallbladder carcinoma progression and resistance to EGFR-targeted therapy through Src kinase activation
Source: Cell Death Differ. 2020 Sep 28;28(3):968–84. doi: 10.1038/s41418-020-00628-4 (PMC7937683; doi:10.1038/s41418-020-00628-4)
Supplement: Supplementary file 16 — Supplementary Table S2 [file 41418_2020_628_MOESM16_ESM.pdf]

Supplementary Table S2. Relationship between EGFR expression and clinicopathological characteristics of gallbladder carcinoma

Variable	Total n=67	EGFR Cytoplasmic (n=11)	EGFR Membranous (n=56)	*P*-value
Age (years)				0.838*
< 65	20	3 (27.3%)	17 (30.4%)	
≥ 65	47	8 (72.7%)	39 (69.6%)	
Gender				0.953*
Male	31	5 (45.5%)	26 (46.4%)	
Female	36	6 (54.5%)	30 (53.6%)	
Pathologic T stage				
1	15	1 (9.1%)	14 (25.0%)	0.832†
2	31	7 (63.6%)	24 (42.9%)	
3	18	3 (27.3%)	15 (26.8%)	
4	3	0 (0.0%)	3 (5.4%)	
Nodal metastasis				0.973*
Absent	49	8 (72.7%)	41 (73.2%)	
Present	18	3 (27.3%)	15 (26.8%)	
Differentiation				0.132†
G 1	10	0 (0.0%)	10 (17.9%)	
G 2	36	6 (54.5%)	30 (53.6%)	
G 3	17	4 (36.4%)	13 (23.2%)	
G 4	4	1 (9.1%)	3 (5.4%)	
Perineural invasion				
Absent	32	4 (36.4%)	28 (50.0%)	0.408*
Present	35	7 (63.6%)	28 (50.0%)	
Lymphatic invasion				
Absent	23	1 (9.1%)	22 (39.3%)	0.054*
Present	44	10 (90.9%)	34 (60.7%)	

*P values were calculated by pairwise comparisons from χ^2 test.

†P values were calculated by comparisons of four groups from linear-by-linear associations.
