# Supplementary material for: Loss of desmoglein-2 promotes gallbladder carcinoma progression and resistance to EGFR-targeted therapy through Src kinase activation
Source: Cell Death Differ. 2020 Sep 28;28(3):968–84. doi: 10.1038/s41418-020-00628-4 (PMC7937683; doi:10.1038/s41418-020-00628-4)
Supplement: Supplementary file 17 — Supplementary Table S3 [file 41418_2020_628_MOESM17_ESM.pdf]

Supplementary Table S3. Univariate and multivariate analyses of the association of prognosis with clinicopathological parameters and cytoplasmic EGFR expression in patients with gallbladder carcinoma

Variables	Univariate		Multivariate	
	HR (95% CI)	*P*	HR (95% CI)	*P*
Age (years, ≥ 65)	0.675 (0.303–1.505)	0.337		
Gender (women)	0.860 (0.426–1.738)	0.674		
Pathologic T stage (T3,4)	3.084 (1.531–6.213)	0.002	2.129 (0.930–4.874)	0.074
Nodal metastasis (yes)	2.967 (1.385–6.355)	0.005	2.556 (1.062–6.151)	0.036
Differentiation (G3,4)	1.529 (1.070–2.186)	0.020	1.619 (1.073–2.443)	0.022
Perineural invasion (yes)	2.006 (0.945–4.258)	0.070		
Lymphatic invasion (yes)	5.232 (1.817–15.063)	0.002	1.726 (0.475–6.274)	0.407
EGFR expression (cytoplasmic)	2.944 (1.242–6.976)	0.014	3.617 (1.354–9.658)	0.010
