# Supplementary material for: Loss of desmoglein-2 promotes gallbladder carcinoma progression and resistance to EGFR-targeted therapy through Src kinase activation
Source: Cell Death Differ. 2020 Sep 28;28(3):968–84. doi: 10.1038/s41418-020-00628-4 (PMC7937683; doi:10.1038/s41418-020-00628-4)
Supplement: Supplementary file 18 — Supplementary Table S4 [file 41418_2020_628_MOESM18_ESM.pdf]

Supplementary Table S4. Relationship between Dsg2 expression and clinicopathological characteristics of gallbladder carcinoma

Variable	Total n=67	Dsg2 HEG (n=37)	Dsg2 LEG (n=30)	*P*-value
Age (years)				0.272*
< 65	20	9 (24.3%)	11 (36.7%)	
≥ 65	47	28 (75.7%)	19 (63.3%)	
Gender				0.354*
Male	31	19 (51.4%)	12 (40.0%)	
Female	36	18 (48.6%)	18 (60.0%)	
Pathologic T stage				
1	15	12 (32.4%)	3 (10.0%)	0.036†
2	31	16 (43.2%)	15 (50.0%)	
3	18	8 (21.6%)	10 (33.3%)	
4	3	1 (2.7%)	2 (6.7%)	
Nodal metastasis				0.602*
Absent	49	28 (75.7%)	21 (70.0%)	
Present	18	9 (24.3%)	9 (30.0%)	
Differentiation				0.684†
G 1	10	6 (16.2%)	4 (13.3%)	
G 2	36	20 (54.1%)	16 (53.3%)	
G 3	17	9 (24.3%)	8 (26.7%)	
G 4	4	2 (5.4%)	2 (6.7%)	
Perineurial invasion				
Abscent	32	22 (59.5%)	10 (33.3%)	0.033*
Present	35	15 (40.5%)	20 (66.7%)	
Lymphatic invasion				
Abscent	23	17 (45.9%)	6 (20.0%)	0.026*
Present	44	20 (54.1%)	24 (80.0%)	

**P* values were calculated by pairwise comparisons from χ^2 test.

†*P* values were calculated by comparisons of four groups from linear-by-linear associations.

HEG, high expression group; LEG low expression group
